# Clinical Outcomes of Tirzepatide or GLP-1 Receptor Agonists in Individuals With Type 2 Diabetes

**DOI:** 10.1001/jamanetworkopen.2024.27258

**Published:** 2024-08-12

**Authors:** Min-Hsiang Chuang, Jui-Yi Chen, Hsien-Yi Wang, Zheng-Hong Jiang, Vin-Cent Wu

**Affiliations:** 1 Division of Nephrology, Department of Internal Medicine, Chi Mei Medical Center, Tainan, Taiwan; 2 Department of Health and Nutrition, Chia Nan University of Pharmacy and Science, Tainan, Taiwan; 3 Department of Sports Management, Chia Nan University of Pharmacy and Science, Tainan, Taiwan; 4 Division of Nephrology, Department of Internal Medicine, National Taiwan University Hospital and National Taiwan University College of Medicine, Taipei

## Abstract

**Importance:**

Despite its demonstrated benefits in improving cardiovascular risk profiles, the association of tirzepatide with mortality and cardiovascular and kidney outcomes compared with glucagon-like peptide 1 receptor agonists (GLP-1 RAs) remains unknown.

**Objective:**

To investigate the association of tirzepatide with mortality and adverse cardiovascular and kidney outcomes compared with GLP-1 RAs in patients with type 2 diabetes.

**Design, Setting, and Participants:**

This retrospective cohort study used US Collaborative Network of TriNetX data collected on individuals with type 2 diabetes aged 18 years or older initiating tirzepatide or GLP-1 RA between June 1, 2022, and June 30, 2023; without stage 5 chronic kidney disease or kidney failure at baseline; and without myocardial infarction or ischemic or hemorrhagic stroke within 60 days of drug initiation.

**Exposures:**

Treatment with tirzepatide compared with GLP-1 RA.

**Main Outcomes and Measures:**

The primary outcome was all-cause mortality, and secondary outcomes included major adverse cardiovascular events (MACEs), the composite of MACEs and all-cause mortality, kidney events, acute kidney injury, and major adverse kidney events. All outcomes were analyzed using Cox proportional hazards regression models.

**Results:**

There were 14 834 patients treated with tirzepatide (mean [SD] age, 55.4 [11.8] years; 8444 [56.9%] female) and 125 474 treated with GLP-1 RA (mean [SD] age, 58.1 [13.3] years; 67 474 [53.8%] female). After a median (IQR) follow-up of 10.5 (5.2-15.7) months, 95 patients (0.6%) in the tirzepatide group and 166 (1.1%) in the GLP-1 RA group died. Tirzepatide treatment was associated with lower hazards of all-cause mortality (adjusted hazard ratio [AHR], 0.58; 95% CI, 0.45-0.75), MACEs (AHR, 0.80; 95% CI, 0.71-0.91), the composite of MACEs and all-cause mortality (AHR, 0.76; 95% CI, 0.68-0.84), kidney events (AHR, 0.52; 95% CI, 0.37-0.73), acute kidney injury (AHR, 0.78; 95% CI, 0.70-0.88), and major adverse kidney events (AHR, 0.54; 95% CI, 0.44-0.67). Treatment with tirzepatide was associated with greater decreases in glycated hemoglobin (treatment difference, −0.34 percentage points; 95% CI, −0.44 to −0.24 percentage points) and body weight (treatment difference, −2.9 kg, 95% CI, −4.8 to −1.1 kg) compared with GLP-1 RA. An interaction test for subgroup analysis revealed consistent results stratified by estimated glomerular filtration rate, glycated hemoglobin level, body mass index, comedications, and comorbidities.

**Conclusions and Relevance:**

In this study, treatment with tirzepatide was associated with lower hazards of all-cause mortality, adverse cardiovascular events, acute kidney injury, and adverse kidney events compared with GLP-1 RA in patients with type 2 diabetes. These findings support the integration of tirzepatide into therapeutic strategies for this population.

## Key Points

**Question:**

What is the association of tirzepatide vs glucagon-like peptide 1 receptor agonist (GLP-1 RA) treatment with all-cause mortality and adverse cardiovascular or kidney events in US patients with type 2 diabetes?

**Findings:**

In this cohort study of 140 308 patients with type 2 diabetes, treatment with tirzepatide was associated with significantly lower hazards of all-cause mortality and major adverse cardiovascular and kidney events compared with GLP-1 RA.

**Meaning:**

These findings suggest that treatment with tirzepatide may confer additional clinical benefits for patients with type 2 diabetes.

## Teaser

This cohort study investigates the risks of mortality and adverse cardiovascular and kidney outcomes among individuals with type 2 diabetes taking tirzepatide vs glucagon-like peptide 1 receptor agonists.

## Introduction

Cardiovascular and kidney diseases are prevalent in patients with diabetes, causing substantial morbidity and mortality.^1,2,3^ Many hypoglycemic agents have been evaluated for cardiovascular and kidney protection beyond their originally designed hypoglycemic effects.^4^ Glucagon-like peptide 1 receptor agonists (GLP-1 RAs) were found to improve kidney and cardiovascular outcomes,^5,6^ with new trials ongoing and results emerging.

Tirzepatide, a dual GLP-1 and glucose-dependent insulinotropic polypeptide (GIP) RA, was developed in view of the synergistic effect of both receptors in achieving negative energy balance besides facilitating glucose-dependent insulin secretion.^7^ Studies have shown tirzepatide’s superiority over semaglutide in reducing glycated hemoglobin (HbA_1c_) and weight (additional reductions of 0.15-0.45 percentage points in HbA_1c_ and 1.9-5.5 kg in body weight) in patients with type 2 diabetes.^8^ Compared with insulin glargine, tirzepatide has been shown to ameliorate annual estimated glomerular filtration rate (eGFR) decline by 2.2 mL/min/1.73 m^2^ and to reduce the urine albumin-to-creatinine ratio by 31.9%.^9,10^ Favorable effects on additional cardiovascular risk factors, including atherogenic lipoproteins and blood pressure, were also observed with tirzepatide compared with semaglutide (increase of 6.8%-7.9% vs 4.4% in high-density lipoprotein cholesterol, decrease of 17.5%-23.7% vs 11.1% in very-low-density lipoprotein cholesterol, and decrease of 4.8-6.5 mm Hg vs 3.6 mm Hg in systolic blood pressure).^8^ However, whether these metabolic improvements lead to benefits in survival and kidney or cardiovascular outcomes remains unclear.

One meta-analysis of randomized clinical trials (RCTs) reported noninferiority of tirzepatide for cardiovascular safety compared with the control groups,^11^ though the included studies were highly heterogenous regarding their comparators (ie, placebo, insulin, GLP-1 RA) and trial participant characteristics, thus precluding a solid conclusion.^11^ Notably, there is a lack of studies directly comparing tirzepatide with GLP-1 RAs, thereby necessitating further investigation in this context. Hence, we investigated the association of tirzepatide with mortality and adverse cardiovascular and kidney outcomes compared with GLP-1 RAs in US patients with type 2 diabetes.

## Method

### Data Source

This cohort study used data from the TriNetX database, which collects deidentified, patient-level data from electronic health records. Information in the TriNetX database comes from health care organizations (HCOs), typically academic health care centers, that collect data from their main and satellite hospitals and outpatient clinics. Available data include demographics, diagnoses (based on *International Classification of Diseases, Tenth Revision, Clinical Modification* codes), procedures (classified by *International Classification of Diseases, Tenth Revision Procedure Coding System* or *Current Procedural Terminology*), medications (Veterans Affairs Drug Classification System and RxNorm codes), laboratory tests (organized using Logical Observation Identifiers Names and Codes), and health care utilization records. We used the US Collaborative Network in TriNetX, which includes data from more than 100 million patients from 62 HCOs in the US.

Patient-level data were analyzed using the built-in statistical tool on the TriNetX platform, which is based on Java, version 11.0.16 (Oracle); R, version 4.0.2 (with packages Hmisc, version 4.1-1 and Survival, version 3.2-3) (R Project for Statistical Computing); and Python, version 3.7 (with the libraries lifelines, version 0.22.4; matplotlib, version 3.5.1; numpy, version 1.21.5; pandas, version 1.3.5; scipy, version 1.7.3; and statsmodels, version, 0.13.2) (Python Software Foundation), with the outputs validated using independent, industry-standard methods. The results were provided to investigators in summarized format. Details regarding the database can be found online^12^ and as previously described.^13,14^

Ethical approval for using the TriNetX database in this study was granted by Chi Mei Hospital’s institutional review board, and institutional review boards from all hospitals involved. Informed consent was waived because this study was conducted using only aggregated statistical summaries of deidentified information. This study was conducted adhering to the principles of the Declaration of Helsinki^15^ and adhered to the Strengthening the Reporting of Observational Studies in Epidemiology (STROBE) reporting guideline.

### Study Population

Patients aged 18 years or older with type 2 diabetes and prescribed tirzepatide or a GLP-1 RA during June 1, 2022, through June 30, 2023, were included because tirzepatide was approved by the US Food and Drug Administration on May 13, 2022. Individuals with acute myocardial infarction, intracranial hemorrhage, or cerebral infarction within 60 days before the index prescription date were excluded to avoid misidentifying incident outcomes. The patients were divided into tirzepatide and GLP-1 RA groups, excluding those previously given medications from the counterpart groups (ie, GLP-1 RA for the tirzepatide group and tirzepatide for the GLP-1 RA group) within 6 months before or any time after the index prescription. To achieve an incident user design, patients with prior use of a GLP-1 RA or tirzepatide before the index date were excluded. To mitigate misinterpretation, individuals with stage 5 chronic kidney disease (CKD), with end-stage kidney disease (ESKD), or receiving dialysis at baseline were excluded from the major adverse kidney event (MAKE) outcome analysis.

We conducted 1:1 propensity score matching (PSM) that involved 48 variables covering demographics, comorbidities, medications, and laboratory results. Patients in both groups were followed for a maximum of 21 months or until data analysis on May 2, 2024. Details regarding the codes used to identify the demographics, diagnoses, procedures, medications, and laboratory results are provided in eTable 1 in Supplement 1.

### Covariates

Covariate selection was guided by clinical relevance, encompassing major comorbidities and risk factors that could contribute to mortality, cardiovascular or kidney outcomes, and baseline health status in accordance with current knowledge. We considered the following variables to adjust for imbalances in baseline characteristics between the tirzepatide and GLP-1 RA groups: age; sex; race and ethnicity (as documented in the electronic health record); cardiovascular disease; dementia; chronic lower-respiratory disease; autoimmune disease; CKD; chronic hepatitis; liver cirrhosis; anemia; HIV; neoplasms; socioeconomic status and psychosocial-related health hazards; diabetic complications; and medicines including diuretics, lipid-lowering agents, hypoglycemic agents, and hypotensive agents. We further adjusted for body mass index (BMI) (calculated as weight in kilograms divided by height in meters squared), systolic blood pressure, hemoglobin, total cholesterol, low-density lipoprotein (LDL) cholesterol, HbA_1c_, and eGFR. Additional details on the categorization and codes adopted to define the covariates are provided in Table 1 and eTable 2 in Supplement 1.

**Table 1.** Baseline Characteristics of the Tirzepatide and GLP-1 RA Groups Before and After Propensity Score Matching

Characteristic	Before matching, No. (%)			After matching, No. (%)		
	Tirzepatide (n = 14 834)	GLP-1 RA (n = 125 474)	Standardized difference	Tirzepatide (n = 14 832)	GLP-1 RA (n = 14 832)	Standardized difference
Age, mean (SD), y	55.4 (11.8)	58.1 (13.3)	0.213	55.4 (11.8)	55.5 (13.3)	0.004
Sex						
Female	8444 (56.9)	67 474 (53.8)	0.064	8444 (56.9)	8436 (56.9)	0.001
Male	5563 (37.5)	53 326 (42.5)	0.106	5563 (37.5)	5577 (37.6)	0.008
Race and ethnicity						
African American or Black	2517 (17.0)	25 212 (20.1)	0.088	2517 (17.0)	2469 (16.6)	0.009
Asian	337 (2.3)	4558 (3.6)	0.080	337 (2.3)	355 (2.4)	0.008
Hispanic or Latinx	957 (6.5)	11 676 (9.3)	0.106	957 (6.5)	906 (6.1)	0.014
White	9978 (67.3)	75 762 (60.4)	0.144	9978 (67.3)	10 052 (67.8)	0.011
Other^a^	466 (3.1)	3137 (2.5)	0.092	466 (3.1)	451 (3.0)	0.003
Unknown	579 (3.9)	5129 (4.1)	0.026	579 (3.9)	599 (4.0)	0.002
Comorbidities						
Hypertension	8265 (55.7)	72 438 (57.7)	0.041	8265 (55.7)	8149 (54.9)	0.016
Ischemic heart disease	1623 (10.9)	16 814 (13.4)	0.075	1623 (10.9)	1588 (10.7)	0.008
Heart failure	821 (5.5)	9447 (7.5)	0.081	821 (5.5)	736 (5.0)	0.026
Atrial fibrillation or flutter	670 (4.5)	7334 (5.8)	0.060	670 (4.5)	685 (4.6)	0.005
Cerebrovascular disease	437 (2.9)	5579 (4.4)	0.080	437 (2.9)	441 (3.0)	0.002
Peripheral vascular disease	320 (2.2)	4150 (3.3)	0.071	320 (2.2)	288 (1.9)	0.015
Chronic lower-respiratory disease	1789 (12.1)	17 673 (14.1)	0.060	1789 (12.1)	1760 (11.9)	0.006
Chronic kidney disease	1261 (8.5)	14 841 (11.8)	0.110	1261 (8.5)	1207 (8.1)	0.013
Anemia	822 (5.5)	10 552 (8.4)	0.113	822 (5.5)	801 (5.4)	0.006
Inflammatory liver disease	308 (2.1)	2064 (1.6)	0.032	308 (2.1)	277 (1.9)	0.015
Liver cirrhosis	259 (1.7)	2241 (1.8)	0.003	259 (1.7)	286 (1.9)	0.014
Neoplasm	2166 (14.6)	19 460 (15.5)	0.025	2166 (14.6)	2209 (14.9)	0.008
Systemic connective tissue disorder	248 (1.7)	2124 (1.7)	0.002	248 (1.7)	244 (1.6)	0.002
Rheumatoid arthritis	74 (0.5)	632 (0.5)	0.001	74 (0.5)	81 (0.5)	0.007
HIV	53 (0.4)	895 (0.7)	0.049	53 (0.4)	45 (0.3)	0.009
Dementia	36 (0.2)	614 (0.5)	0.041	36 (0.2)	32 (0.2)	0.006
Diabetic complications						
Kidney	1281 (8.6)	13700 (10.8)	0.069	1281 (8.6)	1226 (8.3)	0.013
Ophthalmic	699 (4.7)	6015 (4.8)	0.002	698 (4.7)	663 (4.5)	0.011
Neurologic	1610 (10.9)	14143 (11.2)	0.004	1609 (10.8)	1555 (10.5)	0.012
Circulatory	850 (5.7)	7503 (5.9)	0.004	850 (5.7)	812 (5.5)	0.011
SES and psychosocial-related health hazards	322 (2.2)	4019 (3.2)	0.060	322 (2.2)	313 (2.1)	0.004
Medications						
HMG-CoA reductase inhibitors	5530 (37.3)	55 876 (44.5)	0.148	5530 (37.3)	5424 (36.6)	0.015
Fibrates	461 (3.1)	3715 (3.0)	0.009	461 (3.1)	420 (2.8)	0.016
Ezetimibe	427 (2.9)	3536 (2.8)	0.004	427 (2.9)	434 (2.9)	0.003
Biguanides	5449 (36.7)	54 056 (43.1)	0.130	5449 (36.7)	5415 (36.5)	0.005
Sulfonylureas	1397 (9.4)	17 517 (14.0)	0.142	1397 (9.4)	1331 (9.0)	0.015
DPP-4 inhibitors	842 (5.7)	10 329 (8.2)	0.101	842 (5.7)	807 (5.4)	0.010
Thiazolidinediones	393 (2.7)	3565 (2.8)	0.012	393 (2.7)	352 (2.4)	0.018
SGLT2 inhibitors	2544 (17.2)	19 843 (15.8)	0.036	2544 (17.2)	2403 (16.2)	0.026
Insulin	3870 (26.1)	34 311 (27.3)	0.028	3870 (26.1)	3687 (24.9)	0.028
β-Blockers	3082 (20.8)	31 563 (25.2)	0.104	3082 (20.8)	3043 (20.5)	0.006
ACEis	2871 (19.4)	25 812 (20.6)	0.030	2871 (19.4)	2835 (19.1)	0.006
ARBs	2628 (17.7)	27 153 (21.6)	0.099	2628 (17.7)	2578 (17.4)	0.009
Diuretics	3775 (25.5)	35 796 (28.5)	0.069	3775 (25.5)	3706 (25.0)	0.011
Calcium channel blockers	2298 (15.5)	24 569 (19.6)	0.108	2298 (15.5)	2282 (15.4)	0.003
Laboratory tests and examinations						
HbA_1c_, mean (SD), %	7.66 (1.82)	8.01 (1.95)	0.185	7.66 (1.82)	7.69 (1.94)	0.016
≥7%	6391 (43.1)	57 672 (46.0)	0.058	6391 (43.1)	6230 (42.0)	0.022
Hemoglobin, mean (SD), g/dL	13.7 (1.8)	13.4 (1.9)	0.165	13.7 (1.8)	13.6 (1.9)	0.071
≥12 g/dL	7514 (50.7)	62 308 (49.7)	0.020	7514 (50.7)	7451 (50.2)	0.008
eGFR, mean (SD), mL/min/1.73 m^2^	81.4 (24.9)	80.3 (27.7)	0.042	81.4 (24.9)	82.6 (27.4)	0.044
≥45 mL/min/1.73m^2^	9527 (64.2)	79 769 (63.6)	0.014	9527 (64.2)	9330 (62.9)	0.028
SBP, mean (SD), mm Hg	131.4 (17.1)	131.8 (17.9)	0.023	131.4 (17.1)	131.5 (17.7)	0.009
≥130 mm Hg	7591 (51.2)	65 805 (52.4)	0.025	7591 (51.2)	7609 (51.3)	0.002
LDL cholesterol, mean (SD), mg/dL	92.9 (37.3)	92.3 (38.6)	0.016	92.9 (37.3)	93.2 (37.8)	0.008
≥160 mg/dL	563 (3.8)	4833 (3.9)	0.003	563 (3.8)	552 (3.7)	0.004
100-160 mg/dL	3082 (20.8)	24 892 (19.8)	0.023	3082 (20.8)	2984 (20.1)	0.016
Total cholesterol, mean (SD), mg/dL	169.0 (50.8)	169.3 (49.2)	0.006	169.0 (50.8)	169.6 (48.3)	0.014
≥240 mg/dL	697 (4.7)	6617 (5.3)	0.026	697 (4.7)	677 (4.6)	0.006
200–240 mg/dL	1412 (9.5)	13 071 (10.4)	0.030	1412 (9.5)	1394 (9.4)	0.004
BMI, mean (SD)	36.7 (6.2)	34.8 (6.7)	0.285	36.7 (6.2)	36.5 (6.3)	0.026
≥30	8055 (54.3)	58 981 (47.0)	0.146	8055 (54.3)	7980 (53.8)	0.010
25–30	1495 (10.1)	18 674 (14.9)	0.146	1495 (10.1)	1461 (9.9)	0.008

Abbreviations: ACEi, angiotensin-converting enzyme inhibitor; ARB, angiotensin II receptor blocker; BMI, body mass index (calculated as weight in kilograms divided by height in meters squared); DPP-4, dipeptidyl peptidase 4; eGFR, estimated glomerular filtration rate; GLP-1 RA, glucagon-like peptide 1 receptor agonist; HbA_1c_, glycated hemoglobin; HMG-CoA, hydroxymethylglutaryl-CoA; LDL, low-density lipoprotein; SBP, systolic blood pressure; SES, socioeconomic status; SGLT2, sodium-glucose cotransporter 2.

SI conversion factors: To convert HbA_1c_ to proportion of total HbA_1c_, multiply by 0.01; to convert hemoglobin to g/L, multiply by 10; to convert LDL and total cholesterol to mmol/L, multiply by 0.0259.

^a^ Other race and ethnicity included American Indian or Alaska Native, Native Hawaiian or Pacific Islander, multiracial, or not belonging to any racial or ethnic group listed here or in the table.

### Prespecified Outcomes

The primary outcome was all-cause mortality during the follow-up period. Secondary outcomes included major adverse cardiovascular events (MACEs), including myocardial infarction, ischemic or hemorrhagic stroke, or cardiac death; kidney events, including stage 5 CKD, ESKD, or need for dialysis; and MAKEs, including stage 5 CKD, ESKD, need for dialysis, or death.

Additionally, we investigated acute kidney injury (AKI) and the composite of all-cause mortality and MACEs. To establish a baseline for comparison, we included hernia, traumatic intracranial injury, sensorineural hearing loss, skin cancer, and lumbar radiculopathy as negative control outcomes, with selective serotonin reuptake inhibitors and simethicone as negative control exposures. Each component of the composite outcomes was also evaluated for consistency of effect direction. Changes in HbA_1c_ and body weight were evaluated at 4, 8, 12, 16, and 20 months after the index date. The diagnostic, visit, and procedural codes used to define these outcomes are provided in eTable 3 in Supplement 1.

### Subgroup and Sensitivity Analysis

We performed prespecified subgroup analyses based on baseline eGFR (≥45 or <45 mL/min/1.73 m^2^); HbA_1c_ (≥7% or <7%); BMI (≥30 or <30); presence of hypertension, ischemic heart disease, heart failure, or proteinuria; use of metformin, insulin, sodium-glucose cotransporter 2 inhibitors (SGLT2is), angiotensin-converting enzyme inhibitors (ACEis), or angiotensin II receptor blockers (ARBs); or previous GLP-1 RA use in the tirzepatide group. To investigate potential differences based on drug indications, such as glycemic control or weight reduction, we also stratified the patients by HbA_1c_ level and BMI as follows: normal BMI of less than 25 (may have received the medications for diabetes control), BMI of 25 or higher and HbA_1c_ less than 7% (may have been treated for weight loss given that their HbA_1c_ was below the target per current treatment guidelines^16^), and BMI of 25 or higher and HbA_1c_ greater than 7% (may have been treated for both weight loss and glycemic control).

The potential dose effect was evaluated by comparing different tirzepatide doses (maximum ≤7.5 mg or ≥10 mg) with the maximal doses of GLP-1 RA. To investigate the robustness of our findings, a sensitivity analysis was conducted by incorporating individuals without follow-up outcomes into PSM and survival analysis.

### Statistical Analysis

Baseline characteristics of the tirzepatide and GLP-1 RA groups were presented as means with SDs or counts with percentages. Categorical variables were compared by χ^2^ test and continuous variables by independent 2-sample *t* test. One-to-one PSM was performed using the greedy nearest neighbor algorithm with a caliper of 0.1 pooled SDs to balance baseline characteristics between the 2 groups. Variables were considered adequately matched if the between-group standardized difference was less than 0.1. The Kaplan-Meier method was used to calculate survival probabilities after PSM. Adjusted hazard ratios (AHRs) with 95% CIs and *P* values were calculated using Cox proportional hazards regression models for all outcomes. The quantitative effect of unmeasured confounding was analyzed using the E-value method, which assesses the effect magnitude required for unaccounted confounders to elucidate the observed variances between the 2 groups. An E-value of *x* indicates that the observed association could be explained away only by an unmeasured confounder associated with both the treatment and the outcome by a risk ratio of at least *x*-fold each, beyond the measured confounders.^17^ Proportional hazard assumption was tested using Schoenfeld residuals. Missing data regarding the outcomes were addressed by excluding the respective cases. Likewise, patients lost to follow-up were excluded to avoid bias or inaccuracies from incomplete data. All tests were 2-sided with *P* < .05. For the 6 primary and secondary outcomes, Bonferroni correction was applied to account for multiple comparisons, with α set at .0083, providing an overall type I error rate of .0488. Statistical analyses were conducted using the analytic tool on the TriNetX platform and R, version 4.2.2.

## Results

**Figure 1.**
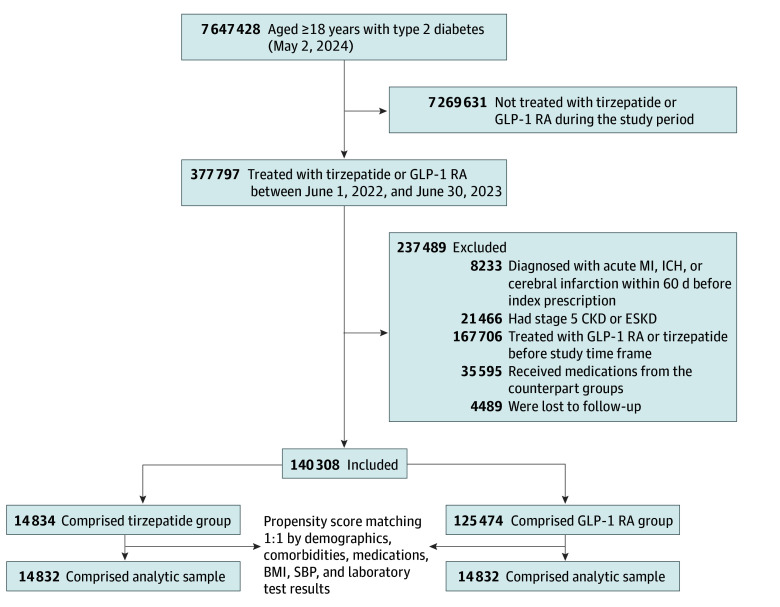
Flow Diagram of Cohort Construction BMI indicates body mass index; CKD, chronic kidney disease; ESKD, end-stage kidney disease; GLP-1 RA, glucagon-like peptide 1 receptor agonist; ICH, intracranial hemorrhage; MI, myocardial infarction; SBP, systolic blood pressure.

Within the cohort of patients with type 2 diabetes, 14 834 received tirzepatide, while 125 474 were prescribed a GLP-1 RA during the study time frame (Figure 1). The numbers of patients excluded due to lack of follow-up after the index date were 348 of 15 182 (2.3%) prescribed tirzepatide and 4141 of 129 615 (3.2%) prescribed a GLP-1 RA. The characteristics of patients lost to follow-up were similar between groups, including younger age, a high proportion of male and Black individuals, high HbA_1c_, and few comorbidities (eTable 4 in Supplement 1). Following PSM, each group included 14 832 individuals for our outcome analyses (Table 1).

Before PSM, tirzepatide-treated patients were younger (mean [SD] age, 55.4 [11.8] years) compared with the GLP-1 RA group (mean [SD] age, 58.1 [13.3] years) (*P* < .001). Additionally, the tirzepatide group had a higher percentage of White patients (67.3% vs 60.4%; *P* < .001), whereas the GLP-1 RA group had higher percentages of Black (20.1% vs 17.0%), Asian (3.6% vs 2.3%), and Hispanic or Latinx (9.3% vs 6.5%) patients; and a higher percentage of female patients (56.9% vs 53.8%; *P* < .001), whereas the GLP-1 RA group a higher percentage of males (42.5% vs 37.5%). Patients in the tirzepatide group also had lower prevalence rates of CKD or anemia, and fewer were using statins, biguanides, sulfonylureas, dipeptidyl peptidase 4 inhibitors, β-blockers, ARBs, or calcium channel blockers. After PSM, the 2 groups were balanced, with standardized differences for all the included covariates being less than 0.1 (Table 1).

### All-Cause Mortality

**Figure 2.**
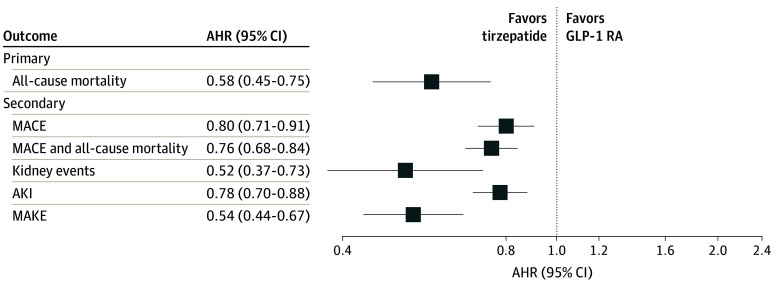
Forest Plot of Primary and Secondary Outcomes AHR indicates adjusted hazard ratio; AKI, acute kidney injury; GLP-1 RA, glucagon-like peptide 1 receptor agonist; MACE, major adverse cardiovascular event; MAKE, major adverse kidney event.

**Figure 3.**
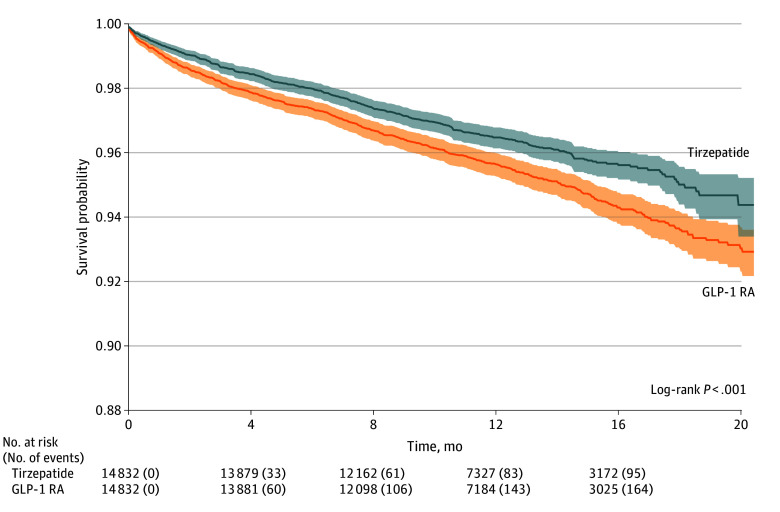
All-Cause Mortality GLP-1 RA indicates glucagon-like peptide 1 receptor agonist.

The overall cohort was followed up for a median of 10.5 months (IQR, 5.2-15.7 months). During the follow-up period, 95 patients (0.6%) in the tirzepatide group and 166 (1.1%) in the GLP-1 RA group died. We observed a lower hazard of all-cause mortality in patients given tirzepatide compared with GLP-1 RA (AHR, 0.58; 95% CI, 0.45-0.75; *P* < .001) (Table 2; Figure 2; Figure 3). The test for proportionality showed no violation of the proportional hazards assumption (Schoenfeld test *P* = .94).

**Table 2.** Comparison of Tirzepatide vs GLP-1 RA for Primary and Secondary Outcomes

Outcome	No. of patients with outcome		AHR (95% CI)	*P* value	E-value (E-value for the upper limit of CI)
	Tirzepatide (n = 14 832)	GLP-1 RA (n = 14 832)			
Primary outcome					
All-cause mortality	95	166	0.58 (0.45-0.75)^a^	<.001^b^	2.82 (1.99)
Secondary outcomes					
MACEs	472	589	0.80 (0.71-0.91)^a^	<.001^b^	1.79 (1.44)
MACE and all-cause mortality	543	721	0.76 (0.68-0.84)^a^	<.001^b^	1.98 (1.65)
Kidney events	53	102	0.52 (0.37-0.73)^a^	<.001^b^	3.24 (2.09)
Acute kidney injury	506	652	0.78 (0.70-0.88)^a^	<.001^b^	1.87 (1.53)
MAKEs	129	241	0.54 (0.44-0.67)^a^	<.001^b^	3.11 (2.35)

Abbreviations: AHR, adjusted hazard ratio; GLP-1 RA, glucagon-like peptide 1 receptor agonist; MACE, major adverse cardiovascular event; MAKE, major adverse kidney event.

^a^ Proportionality (Schoenfeld test *P* > .05).

^b^ Bonferroni-corrected α* = *.0083.

### Secondary Outcomes

The hazard of MACEs was lower among patients treated with tirzepatide (AHR, 0.80; 95% CI, 0.71-0.91; *P* < .001), as was the hazard of kidney events (AHR, 0.52; 95% CI, 0.37-0.73; *P* < .001), AKI (AHR, 0.78; 95% CI, 0.70-0.88; *P* < .001), MAKEs (AHR, 0.54; 95% CI, 0.44-0.67; *P* < .001), and the composite of MACEs and all-cause mortality (AHR, 0.76; 95% CI, 0.68-0.84; *P* < .001), favoring tirzepatide (Table 2; Figure 2). Our E-value analysis indicated an unlikely significant influence from unmeasured confounders (E-values of the point estimates [upper limits of the CI] for all-cause mortality, MACEs, and kidney events were 2.82 [1.99], 1.79 [1.44], and 3.24 [2.09], respectively) (Table 2). The hazard of each MACE component (ie, myocardial infarction, ischemic or hemorrhagic stroke, cardiac death) and MAKE component (ie, stage 5 CKD or ESKD) was consistently lower with tirzepatide compared with GLP-1 RAs, with AHRs ranging from 0.78 (95% CI, 0.67-0.91) to 0.91 (95% CI, 0.56-1.45) for MACE components and from 0.45 (95% CI, 0.25-0.82) to 0.49 (95% CI, 0.35-0.67) for MAKE components (eTable 5 in Supplement 1). Sensitivity analysis incorporating patients without any follow-up into PSM and survival analyses showed consistent results with our main analysis (eTable 6 in Supplement 1).

Compared with GLP-1 RA treatment, tirzepatide treatment was associated with greater reductions in HbA_1c_ (treatment difference, −0.34 percentage points; 95% CI, −0.44 to −0.24 percentage points) and body weight (treatment difference, −2.9 kg; 95% CI, −4.8 to −1.1 kg) over 20 months. The most significant HbA_1c_ decrease occurred within the first 4 months and stabilized after 8 months, while weight reduction persisted through 16 to 20 months (eTable 7 and eFigure 1 in Supplement 1).

Results comparing tirzepatide vs maximal doses of GLP-1 RA closely matched the main analysis, with similar AHRs and corresponding 95% CIs (eFigure 2A in Supplement 1). When stratified by tirzepatide dose, treatment with 10 mg or more was associated with lower hazards of all-cause mortality, MACEs, and MAKEs compared with 7.5 mg or less (eFigure 2B and C in Supplement 1).

### Negative Controls and Subgroup Analysis

Our study revealed no significant differences in the hazard of negative control outcomes between the tirzepatide and GLP-1 RA groups for hernia, traumatic intracranial injury, sensorineural hearing loss, lumbar radiculopathy, and skin cancer. Results were consistent when selective serotonin reuptake inhibitors and simethicone were introduced as negative control exposures (eFigure 3 in Supplement 1). The interaction test revealed no significant effect differences across all subgroups stratified by eGFR; HbA_1c_; presence of hypertension, ischemic heart disease, heart failure, proteinuria, or diabetic complications; use of metformin, insulin, SGLT2is, ACEis, or ARBs; previous use of GLP-1 RAs; potential treatment indications (body weight loss, glycemic control, or both); LDL cholesterol level; or BMI (all interaction test *P* > .05) (eFigures 4-9 in Supplement 1).

## Discussion

From this retrospective cohort study of 140 308 US patients with type 2 diabetes treated with tirzepatide or GLP-1 RAs, we have for the first time to our knowledge presented evidence substantiating that tirzepatide therapy is associated with lower risks of all-cause mortality, MACEs, MAKEs, and AKI compared with GLP-1 RA therapy after a median follow-up of 10.5 months. Consistent results were found in subgroup analyses stratified by eGFR; HbA_1c_; presence of cardiovascular disease, proteinuria, or diabetic complications; use of metformin, insulin, SGLT2is, ACEis, or ARBs; previous GLP-1 RA treatment; potential indications; LDL cholesterol level; and BMI.

The association of tirzepatide with less all-cause mortality and MACEs may be attributable to tirzepatide’s efficacy in improving the cardiovascular risk profile, including blood pressure, LDL, triglycerides, HbA_1c_,^8,18^ cardiovascular risk biomarkers,^19^ and body weight in patients with obesity.^18,20^ Consistently, a network meta-analysis comparing various GLP-1 RAs and dual and triple receptor agonists showed that tirzepatide was the most effective in reducing HbA_1c_, fasting glucose, triglyceride level, and waist circumference compared with all other GLP-1 RAs.^21^ The meta-analysis also ranked tirzepatide second for weight loss, surpassed only by cagrilintide-semaglutide, another dual receptor agonist under development. Our analysis similarly showed an association of greater HbA_1c_ and weight loss with tirzepatide, and the trajectories aligned with those reported in previous trials,^8^ suggesting a potential mediatory role for these factors. This finding is in line with a recent report in which withdrawing tirzepatide led to substantial weight regain.^22^ Nonetheless, studies on the efficacy of tirzepatide in reducing adverse cardiovascular outcomes have yielded inconsistent results, suggesting that GIP may have both beneficial^23,24^ and untoward cardiovascular effects,^25,26^ though the results may be due to reverse causality.

While 1 meta-analysis of 7 RCTs reported no significant difference in all-cause mortality, cardiovascular mortality, and MACE risk between tirzepatide and a mix of comparators, including placebo, 2 insulin formulations, and GLP-1 RAs,^11^ another study of 8 RCTs contrarily showed a significant decrease in all-cause mortality and MACE risk with tirzepatide treatment compared with the control group receiving placebo or active comparators.^27^ The disparity may be due to differences in the included studies, comparators, sample sizes, baseline patient characteristics, and outcome definitions. Both studies carried high clinical heterogeneity in participant inclusion criteria and comparator selection. Of note, previous trials on tirzepatide and GLP-1 RAs for cardiovascular and mortality outcomes had median follow-ups of 1.6 to 3.8 years,^28,29^ but the survival curves for composite primary cardiovascular outcomes began diverging as early as 8 weeks to approximately 6 months,^28,29,30,31^ suggesting potential early benefits after treatment initiation.

Both GLP-1 and GIP belong to the incretin system that regulates hormone secretion in response to carbohydrate intake.^32^ Although the effect of GIP has been found to be greatly attenuated in patients with type 2 diabetes,^33^ subsequent research has shown that islet β-cell sensitivity to GIP may be restored with glycemic control.^34^ Glucose-dependent insulinotropic polypeptide acts in the central nervous system by blocking nausea and emesis from GLP-1 agonism without affecting satiety or energy intake reduction,^35^ thereby improving overall treatment tolerability. In animal studies, dual GLP-1 and GIP coagonists synergistically reduced fat mass and improved metabolic profiles superior to selective GLP-1 agonist, contrary to selective GIP agonist, which was weight neutral.^36^ Subsequent clinical trials confirmed that tirzepatide was more effective than selective GLP-1 agonists in increasing insulin secretion and sensitivity, improving meal tolerance, and suppressing glucagon secretion,^37^ with similar suppression of appetite and calorie intake.^38^ Additionally, nutrition-independent regulation of the GIP pathway through inflammatory stimuli may stabilize atherosclerosis plaques by inhibiting monocyte and macrophage activation^39^ and suppressing cardiomyocyte enlargement, apoptosis, and interstitial fibrosis.^40^ The potential superiority of GIP and GLP-1 RAs over GLP-1 RAs for MACEs and mortality, as suggested by our findings, indicates that combined activation of GIP and GLP-1 receptors may provide additional benefits over the activation of GLP-1 receptors alone.

The kidney-protective effect of tirzepatide was first shown in post hoc analyses of the SURPASS-4 (Tirzepatide vs Insulin Glargine in Type 2 Diabetes and Increased Cardiovascular Risk) trial, which recruited patients with type 2 diabetes, overweight, and established or high risk for cardiovascular disease. Patients treated with tirzepatide added to insulin had less eGFR decline^10,41^ and proteinuria progression and fewer composite kidney end points compared with the insulin group.^10^ These findings align with studies on selective GLP-1 agonists.^42^ Notably, our study is the first, to our knowledge, to directly compare the association with kidney outcomes between tirzepatide and selective GLP-1 RAs. Preclinical studies suggested that GIP may attenuate local adipose tissue inflammation and reduce proinflammatory cytokine levels,^43^ which may act favorably on the pathogenesis of diabetic kidney disease.^44^ On the other hand, GLP-1 receptors are present on preglomerular vascular smooth muscle, glomerular, and tubular cells and, thus, may directly affect the kidney.^45,46^ Proposed mechanisms include afferent arteriole vasodilation, proximal tubular diuresis, and inhibition of glomerular hyperfiltration.^45,47^ Importantly, pharmacokinetic studies showed that tirzepatide exposure was not significantly affected by kidney function impairment^48^ and represented a viable therapy for patients with stage 4 and 5 CKD, for whom few options existed.^49^ Treatment with tirzepatide or a GLP-1 RA might pose a risk of AKI due to dehydration from gastrointestinal adverse effects.^50,51^ Although our analysis found a lower hazard of AKI in the tirzepatide group compared with the GLP-1 RA group, this result should be interpreted with caution since we could not identify the cause of these AKI events or their chronologic association with tirzepatide or GLP-1 RA use.

The interaction test from subgroup analyses revealed no significant discrepancies in our primary and secondary outcomes among different subgroups. Of note, most patients included in this study were relatively healthy, with an eGFR of at least 45 mL/min/1.73 m^2^; no history of ischemic heart disease or heart failure; and not taking metformin, insulin, SGLT2is, ACEis, or ARBs. Accordingly, the null results from our subgroup analyses may be partly due to reduced statistical power from smaller sample sizes. Nevertheless, for individuals already benefiting from efficacious treatments, the integration of GIP agonists surpassed the efficacy of existing therapeutic regimens in specific combinations. The potential dose-response relationships observed in our analysis stratified by tirzepatide dose also support tirzepatide’s possible efficacy.

Our findings also provide directions for future research in other target populations. The observational nature of our data precluded inference of causality, which might be confirmed in future research, such as in ongoing RCTs evaluating the efficacy of tirzepatide in improving cardiovascular and kidney outcomes compared with GLP-1 agonists^52^ and in treating patients with heart failure with preserved ejection fraction.^53^

### Limitations

This study has some limitations. First, data from TriNetX were registry based, and therefore, misidentification and underrepresentation of mild cases or of individuals not engaged with the health care system may exist and influence the results. The reliance on diagnostic codes to identify variables and outcomes may also result in misclassification, potentially biasing the results. To address this information bias, we conducted specificity tests comparing unrelated events between the tirzepatide and GLP-1 RA groups and found no significant differences, indicating little registration bias. Second, the duration of treatment could not be ascertained from the database, precluding analysis of a long-term carryover effect. While we observed clinical benefits, their effect sizes were relatively imprecise when interpreting the confidence intervals and require further investigation. Third, unmeasured variables may have contributed to the studied outcomes, introducing additional confounding. Given the observational nature of this study and that some baseline characteristics differed between the groups, residual confounding cannot be eliminated entirely. To address potential bias, we juxtaposed unrelated events among GLP-1 RA and tirzepatide users, finding no discernible disparities and indicating that bias from residual confounding may be negligible. We also used comedication and biochemical results as proxies for disease severity to mitigate potential confounding. Higher E-values that surpassed the hazard ratios indicated that minor unaccounted confounders could not counteract the observed association, thus increasing the potential for causality. Fourth, although consistent results were found in sensitivity analyses incorporating individuals lost to follow-up into survival analysis, much of the nonsummarized data and their changes over time were restricted from investigators to safeguard the detailed health information from potential misuse in identifying and tracking specific individuals, thereby precluding further imputation methods. Fifth, information directly related to socioeconomic status, including education, income, and insurance status, was not documented, limiting further investigation into their contributions. Finally, this study comprised patients with type 2 diabetes, of whom most were relatively healthy without underlying cardiovascular or advanced kidney diseases, so the applicability of our findings to other clinical settings remain to be explored.

## Conclusions

In summary, this cohort study provided evidence supporting the association of tirzepatide treatment, compared to GLP-1 RAs, with lower hazards of all-cause mortality and adverse cardiovascular or kidney events through a head-to-head comparison in patients with type 2 diabetes. These insights advocate for the integration of tirzepatide into therapeutic strategies for managing type 2 diabetes and highlight its potential to enhance current clinical practice.

## Notes

Supplement 1. **eTable 1.** Demographic, Diagnostic, Procedural, Medication, Visit, and Laboratory Codes Used in the Definition of the Cohorts **eTable 2.** Demographic, Diagnostic, and Laboratory Codes Used in the Definition of Covariates **eTable 3.** Diagnostic, Visit, and Procedural Codes Used in the Definition of Outcomes **eTable 4.** Numbers and Characteristics of Individuals Excluded Because of a Lack of Any Follow-Up **eTable 5.** Adjusted Hazard Ratios for the Components of Major Adverse Cardiovascular Events and Kidney Events **eTable 6.** Sensitivity Analysis Incorporating Individuals Without Follow-Up Into Propensity Score Matching and Survival Analysis of Primary and Secondary Outcomes **eTable 7.** Changes in Mean Glycated Hemoglobin Level and Body Weight (95% CI) **eFigure 1.** Changes in Glycated Hemoglobin Level and Body Weight From Baseline **eFigure 2.** Forest Plot of Primary and Secondary Outcomes Compared Between Different Doses of Tirzepatide and GLP-1 RA **eFigure 3.** Results for Negative Control Outcomes and Analysis With Negative Control Exposure **eFigure 4.** Subgroup Analysis of All-Cause Mortality Between Tirzepatide and GLP-1 RA Groups **eFigure 5.** Subgroup Analysis of Major Adverse Cardiovascular Events Between Tirzepatide and GLP-1 RA Groups **eFigure 6.** Subgroup Analysis of the Composite of Major Adverse Cardiovascular Events and All-Cause Mortality Between Tirzepatide and GLP-1 RA Groups **eFigure 7.** Subgroup Analysis of Kidney Events Between Tirzepatide and GLP-1 RA Groups **eFigure 8.** Subgroup Analysis of Acute Kidney Injury Between Tirzepatide and GLP-1 RA Groups **eFigure 9.** Subgroup Analysis of Major Adverse Kidney Events Between Tirzepatide and GLP-1 RA Groups

Supplement 2.
Data Sharing Statement
