# Supplementary material for: Clinical Outcomes of Tirzepatide or GLP-1 Receptor Agonists in Individuals With Type 2 Diabetes
Source: JAMA Netw Open. 2024 Aug 12;7(8):e2427258. doi: 10.1001/jamanetworkopen.2024.27258 (PMC11320168; doi:10.1001/jamanetworkopen.2024.27258)
Supplement: Supplement 1. — eTable 1. Demographic, Diagnostic, Procedural, Medication, Visit, and Laboratory Codes Used in the Definition of the Cohorts eTable 2. Demographic, Diagnostic, and Laboratory Codes Used in the Definition of Covariates eTable 3. Diagnostic, Visit, and Procedural Codes Used in the Definition of Outcomes eTable 4. Numbers and Characteristics of Individuals Excluded Because of a Lack of Any Follow-Up eTable 5. Adjusted Hazard Ratios for the Components of Major Adverse Cardiovascular Events and Kidney Events eTable 6. Sensitivity Analysis Incorporating Individuals Without Follow-Up Into Propensity Score Matching and Survival Analysis of Primary and Secondary Outcomes eTable 7. Changes in Mean Glycated Hemoglobin Level and Body Weight (95% CI) eFigure 1. Changes in Glycated Hemoglobin Level and Body Weight From Baseline eFigure 2. Forest Plot of Primary and Secondary Outcomes Compared Between Different Doses of Tirzepatide and GLP-1 RA eFigure 3. Results for Negative Control Outcomes and Analysis With Negative Control Exposure eFigure 4. Subgroup Analysis of All-Cause Mortality Between Tirzepatide and GLP-1 RA Groups eFigure 5. Subgroup Analysis of Major Adverse Cardiovascular Events Between Tirzepatide and GLP-1 RA Groups eFigure 6. Subgroup Analysis of the Composite of Major Adverse Cardiovascular Events and All-Cause Mortality Between Tirzepatide and GLP-1 RA Groups eFigure 7. Subgroup Analysis of Kidney Events Between Tirzepatide and GLP-1 RA Groups eFigure 8. Subgroup Analysis of Acute Kidney Injury Between Tirzepatide and GLP-1 RA Groups eFigure 9. Subgroup Analysis of Major Adverse Kidney Events Between Tirzepatide and GLP-1 RA Groups [file jamanetwopen-e2427258-s001.pdf]

Supplemental Online Content

Chuang M-H, Chen J-Y, Wang H-Y, Jiang Z-H, Wu V-C. Clinical outcomes of tirzepatide or GLP-1 receptor agonists in individuals with type 2 diabetes. *JAMA Netw Open*. 2024;7(8):e2427258. doi:10.1001/jamanetworkopen.2024.27258

eTable 1. Demographic, Diagnostic, Procedural, Medication, Visit, and Laboratory Codes Used in the Definition of the Cohorts

eTable 2. Demographic, Diagnostic, and Laboratory Codes Used in the Definition of Covariates

eTable 3. Diagnostic, Visit, and Procedural Codes Used in the Definition of Outcomes

eTable 4. Numbers and Characteristics of Individuals Excluded Because of a Lack of Any Follow-Up

eTable 5. Adjusted Hazard Ratios for the Components of Major Adverse Cardiovascular Events and Kidney Events

eTable 6. Sensitivity Analysis Incorporating Individuals Without Follow-Up Into Propensity Score Matching and Survival Analysis of Primary and Secondary Outcomes

eTable 7. Changes in Mean Glycated Hemoglobin Level and Body Weight (95% CI)

eFigure 1. Changes in Glycated Hemoglobin Level and Body Weight From Baseline

eFigure 2. Forest Plot of Primary and Secondary Outcomes Compared Between Different Doses of Tirzepatide and GLP-1 RA

eFigure 3. Results for Negative Control Outcomes and Analysis With Negative Control Exposure

eFigure 4. Subgroup Analysis of All-Cause Mortality Between Tirzepatide and GLP-1 RA Groups

eFigure 5. Subgroup Analysis of Major Adverse Cardiovascular Events Between Tirzepatide and GLP-1 RA Groups

eFigure 6. Subgroup Analysis of the Composite of Major Adverse Cardiovascular Events and All-Cause Mortality Between Tirzepatide and GLP-1 RA Groups

eFigure 7. Subgroup Analysis of Kidney Events Between Tirzepatide and GLP-1 RA Groups

eFigure 8. Subgroup Analysis of Acute Kidney Injury Between Tirzepatide and GLP-1 RA Groups

eFigure 9. Subgroup Analysis of Major Adverse Kidney Events Between Tirzepatide and GLP-1 RA Groups

This supplemental material has been provided by the authors to give readers additional information about their work.

© 2024 Chuang M-H et al. *JAMA Network Open.*

eTable 1. Demographic, Diagnostic, Procedural, Medication, Visit, and Laboratory Codes Used in the Definition of the Cohorts

Category	Code	Description
**Tirzepatide group**		
**#1**: At least 18 years old		
Demographics	Age	Age (at least 18 years)
**#2**: Patients with type 2 diabetes mellitus treated with tirzepatide but not GLP-1 receptor agonists. (# 2.2 must be fulfilled within 6 months before or any time after #2.1)		
**#2.1**: Patients with Type 2 diabetes mellitus treated with tirzepatide (must have both of the following) Date constraint: the terms in this group occurred between Jun 1, 2022 and Jun 30, 2023		
Diagnosis	UMLS:ICD10CM:E11	Type 2 diabetes mellitus
Medication	NLM:RXNORM:2601723	Tirzepatide
**#2.2**: Not receiving GLP-1 receptor agonists (cannot have the following)		
medication	NLM:ATC:A10BJ	Glucagon-like peptide-1 (GLP-1) analogues
**#3**: Without recent major adverse cardiovascular events (cannot have any of the following) #3 must be fulfilled within 60 days on or before #2.1		
Diagnosis	UMLS:ICD10CM:I21	Acute myocardial infarction
Diagnosis	UMLS:ICD10CM:I22	Subsequent ST elevation (STEMI) and non-ST elevation (NSTEMI) myocardial infarction
Diagnosis	UMLS:ICD10CM:I61	Nontraumatic intracerebral hemorrhage
Diagnosis	UMLS:ICD10CM:I62	Other and unspecified nontraumatic intracranial hemorrhage
Diagnosis	UMLS:ICD10CM:I63	Cerebral infarction
#4: Incident user (cannot have the following) #4 must be fulfilled before #2.1		
Medication	NLM:RXNORM:2601723	Tirzepatide
**GLP-1 receptor agonist group**		
**#1**: At least 18 years old		
Demographics	Age	Age (at least 18 years)
**#2**: Patients with type 2 diabetes mellitus treated with tirzepatide but not GLP-1 receptor agonists. (# 2.2 must be fulfilled within 6 months before or any time after #2.1)		
**#2.1**: Patients with Type 2 diabetes mellitus treated with tirzepatide (must have both of the following; Date constraint: the terms in this group occurred between Jun 1, 2022 and Jun 30, 2023)		
Diagnosis	UMLS:ICD10CM:E11	Type 2 diabetes mellitus
Medication	NLM:ATC:A10BJ	Glucagon-like peptide-1 (GLP-1) analogues
**#2.2**: Not receiving GLP-1 receptor agonists (cannot have the following)		
Medication	NLM:RXNORM:2601723	Tirzepatide
**#3**: Without recent major adverse cardiovascular events (cannot have any of the following) #3 must be fulfilled within 60 days on or before #2.1		
Diagnosis	UMLS:ICD10CM:I21	Acute myocardial infarction
Diagnosis	UMLS:ICD10CM:I22	Subsequent ST elevation (STEMI) and non-ST elevation (NSTEMI) myocardial infarction
Diagnosis	UMLS:ICD10CM:I61	Nontraumatic intracerebral hemorrhage
Diagnosis	UMLS:ICD10CM:I62	Other and unspecified nontraumatic intracranial hemorrhage
Diagnosis	UMLS:ICD10CM:I63	Cerebral infarction
#4: Incident user (cannot have the following) #4 must be fulfilled before #2.1		
Medication	NLM:ATC:A10BJ	Glucagon-like peptide-1 (GLP-1) analogues

© 2024 Chuang M-H et al. *JAMA Network Open.*

eTable 2. Demographic, Diagnostic, and Laboratory Codes Used in the Definition of Covariates

Category	Code	Description
Demographics	AI	Age at index
Demographics	F	Female
Demographics	2106-3	White
Demographics	2054-5	Black or African American
Demographics	2028-9	Asian
Diagnosis	E08-E13	Diabetes mellitus
Diagnosis	I10-I16	Hypertensive diseases
Diagnosis	C00-D49	Neoplasms
Diagnosis	J40-J47	Chronic lower respiratory diseases
Diagnosis	I20-I25	Ischemic heart diseases
Diagnosis	N18	Chronic kidney disease (CKD)
Diagnosis	D60-D64	Aplastic and other anemias
Diagnosis	I50	Heart failure
Diagnosis	I48	Atrial fibrillation and flutter
Diagnosis	I60-I69	Cerebrovascular diseases
Diagnosis	I73.9	Peripheral vascular disease, unspecified
Diagnosis	K75	Other inflammatory liver diseases
Diagnosis	K74	Fibrosis and cirrhosis of liver
Diagnosis	M30-M36	Systemic connective tissue disorders
Diagnosis	M05	Rheumatoid arthritis with rheumatoid factor
Diagnosis	B20-B20	Human immunodeficiency virus (HIV) disease
Diagnosis	F03	Unspecified dementia
Diagnosis	E11.2	Type 2 diabetes mellitus with kidney complications
Diagnosis	E11.3	Type 2 diabetes mellitus with ophthalmic complications
Diagnosis	E11.4	Type 2 diabetes mellitus with neurological complications
Diagnosis	E11.5	Type 2 diabetes mellitus with circulatory complications
Diagnosis	Z55-Z65	Health hazards related to socioeconomic and psychosocial circumstances
Medication	C10AA	HMG CoA reductase inhibitors
Medication	A10BA	Biguanides
Medication	C03	Diuretics
Medication	A10A	Insulins and analogues
Medication	C07	Beta blocking agents
Medication	C09C	Angiotensin II receptor blockers
Medication	C09A	Ace inhibitors
Medication	A10BK	Sodium-glucose co-transporter 2 (SGLT2) inhibitors
Medication	C08	Calcium channel blockers
Medication	A10BB	Sulfonylureas
Medication	A10BH	Dipeptidyl peptidase 4 (DPP-4) inhibitors
Medication	C10AB	Fibrates
Medication	A10BG	Thiazolidinediones
Medication	341248	Ezetimibe
Laboratory	8001	Glomerular filtration rate by creatinine-based formula
Laboratory	9037	Hemoglobin A1c/hemoglobin in blood

© 2024 Chuang M-H et al. *JAMA Network Open.*

Category	Code	Description
Laboratory	9014	Hemoglobin in blood
Laboratory	9085	Blood pressure, systolic
Laboratory	9002	Cholesterol in LDL in serum or plasma
Laboratory	9000	Cholesterol in serum or plasma
Laboratory	9083	Body mass index (BMI)

© 2024 Chuang M-H et al. *JAMA Network Open.*

eTable 3. Diagnostic, Visit, and Procedural Codes Used in the Definition of Outcomes

**Category**	**Code**	**Description**
**#1**: All-cause mortality (have any of the following)		
Demographics	Deceased	Deceased
Diagnosis	UMLS:ICD10CM:R99	Ill-defined and unknown cause of mortality
**#2**: Major adverse cardiovascular events (have any of the following)		
Diagnosis	UMLS:ICD10CM:I21	Acute myocardial infarction
Diagnosis	UMLS:ICD10CM:I46.2	Cardiac arrest due to underlying cardiac condition
Diagnosis	UMLS:ICD10CM:I46.9	Cardiac arrest, cause unspecified
Diagnosis	UMLS:ICD10CM:I63	Cerebral infarction
Diagnosis	UMLS:ICD10CM:I61	Nontraumatic intracerebral hemorrhage
Diagnosis	UMLS:ICD10CM:I22	Subsequent ST elevation (STEMI) and non-ST elevation (NSTEMI) myocardial infarction
Diagnosis	UMLS:ICD10CM:I62	Other and unspecified nontraumatic intracranial hemorrhage
**#3**: Kidney events (have any of the following)		
Diagnosis	UMLS:ICD10CM:N18.5	Chronic kidney disease, stage 5
Diagnosis	UMLS:ICD10CM:N18.6	End stage renal disease
Diagnosis	UMLS:ICD10CM:Z99.2	Dependence on renal dialysis
Diagnosis	UMLS:ICD10CM:N19	Unspecified kidney failure
Procedure	UMLS:CPT:1012740	Dialysis Services and Procedures
Procedure	UMLS:CPT:1029674	Dialysis Circuit Procedures
**#4**: Acute kidney injury (have any of the following)		
Diagnosis	UMLS:ICD10CM:N17	Acute kidney failure
**#5**: Major adverse cardiovascular event or all-cause mortality (have #1 or #2)		
**#6**: Major adverse kidney event (have #1 or #3)		
**#7**: Glycated hemoglobin (HbA1c) level		
Laboratory	TNX:9037	Hemoglobin A1c/hemoglobin in blood
**#8**: Body weight		
Laboratory	TNX:9081	Body weight
**#9**: Negative control outcomes		
**#9.1**: Hernia, excluding patients with outcome prior to the follow-up time window		
Diagnosis	UMLS:ICD10CM:K40-K46	Hernia
**#9.2**: Traumatic brain injury, excluding patients with outcome prior to the follow-up time window		
Diagnosis	UMLS:ICD10CM:S06.5	Traumatic subdural hemorrhage
Diagnosis	UMLS:ICD10CM:S06.6	Traumatic subarachnoid hemorrhage
Diagnosis	UMLS:ICD10CM:S06.2	Diffuse traumatic brain injury
Diagnosis	UMLS:ICD10CM:S06.3	Focal traumatic brain injury
**#9.3**: Lumbar radiculopathy, excluding patients with outcome prior to the follow-up time window		
Diagnosis	UMLS:ICD10CM:M54.16	Radiculopathy, lumbar region
**#9.4**: Sensorineural hearing loss, excluding patients with outcome prior to the follow-up time window		
Diagnosis	UMLS:ICD10CM:H90	Conductive and sensorineural hearing loss
**#9.5**: Skin cancer, excluding patients with outcome prior to the follow-up time window		
Diagnosis	UMLS:ICD10CM:C44	Other and unspecified malignant neoplasm of skin

© 2024 Chuang M-H et al. *JAMA Network Open.*

eTable 4. Numbers and Characteristics of Individuals Excluded Because of a Lack of any Follow-Up

Variables	GLP-1 receptor agonist, No (%)		Tirzepatide, No (%)	
	Included	No follow-up	Included	No follow-up
**Number of patients**	125,474	4,141	14,834	348
**Demographics**				
Age, mean (SD), y	58.1 (13.3)	56 (13.7)***	55.4 (11.8)	51.8 (13.0)***
Female	67,474 (53.8)	2,127 (51.4)**	8,444 (56.9)	179 (51.3)*
African American/Black	25,212 (20.1)	992 (24.0)***	2,517 (17.0)	73 (20.9)**
Asian	4,558 (3.6)	149 (3.6)	337 (2.3)	≤10 (≤ 2.9)†
Hispanic/Latinx	11,676 (9.3)	320 (7.7)**	957 (6.5)	19 (5.4)
White	75,762 (60.4)	2,251 (54.4)***	9,978 (67.3)	206 (59.0)**
**Comorbidities**				
Hypertensive diseases	72,438 (57.7)	1,383 (33.4)***	8,265 (55.7)	87 (24.9)***
Ischemic heart diseases	16,814 (13.4)	295 (7.1)***	1,623 (10.9)	≤10 (≤ 2.9)†
Heart failure	9,447 (7.5)	161 (3.9)***	821 (5.5)	≤10 (≤ 2.9)†
Atrial fibrillation or flutter	7,334 (5.8)	120 (2.9)***	670 (4.5)	≤10 (≤ 2.9)†
Cerebrovascular diseases	5,579 (4.4)	86 (2.1)***	437 (2.9)	≤10 (≤ 2.9)†
Peripheral vascular diseases	4,150 (3.3)	51 (1.2)***	320 (2.2)	≤10 (≤ 2.9)†
Chronic lower respiratory diseases	17,673 (14.1)	264 (6.4)***	1,789 (12.1)	≤10 (≤ 2.9)†
Chronic kidney disease	14,841 (11.8)	253 (6.1)***	1,261 (8.5)	15 (4.3)**
Anemia	10,552 (8.4)	181 (4.4)***	822 (5.5)	≤10 (≤ 2.9)†
Inflammatory liver diseases	2,064 (1.6)	17 (0.4)***	308 (2.1)	≤10 (≤ 2.9)†
Liver cirrhosis	2,241 (1.8)	26 (0.6)***	259 (1.7)	0 (0.0)*
Neoplasms	19,460 (15.5)	227 (5.5)***	2,166 (14.6)	16 (4.6)***
Systemic connective tissue disorders	2,124 (1.7)	17 (0.4)***	248 (1.7)	≤10 (≤ 2.9)†
Rheumatoid arthritis	632 (0.5)	≤10 (≤ 0.2)†	74 (0.5)	≤10 (≤ 2.9)†
HIV disease	895 (0.7)	≤10 (≤ 0.2)†	53 (0.4)	0 (0.0)
Dementia	614 (0.5)	17 (0.4)	36 (0.2)	0 (0.0)
Diabetic complications				
Kidney complications	13,700 (10.8)	302 (7.3)***	1,281 (8.6)	15 (4.3)***
Ophthalmic complications	6,015 (4.8)	77 (1.9)***	699 (4.7)	≤10 (≤ 2.9)†
Neurological complications	14,143 (11.2)	223 (5.4)***	1,610 (10.9)	11 (3.2)***
Circulatory complications	7,503 (5.9)	129 (3.1)***	850 (5.7)	≤10 (≤ 2.9)†
SES and psychosocial-related hazards	4,019 (3.2)	58 (1.4)***	322 (2.2)	≤10 (≤ 2.9)†
**Medication**				
HMG CoA reductase inhibitors	55,876 (44.5)	1,274 (30.8)***	5,530 (37.3)	79 (22.6)***
Fibrates	3,715 (3.0)	93 (2.2)**	461 (3.1)	≤10 (≤ 2.9)†
Ezetimibe	3,536 (2.8)	61 (1.5)***	427 (2.9)	≤10 (≤ 2.9)†
Biguanides	54,056 (43.1)	1,184 (28.6)***	5,449 (36.7)	74 (21.2)***
Sulfonylureas	17,517 (14.0)	426 (10.3)***	1,397 (9.4)	23 (6.6)
DPP-4 inhibitors	10,329 (8.2)	228 (5.5)***	842 (5.7)	14 (4.0)
Thiazolidinediones	3,565 (2.8)	87 (2.1)**	393 (2.7)	≤10 (≤ 2.9)†
SGLT2 inhibitors	19,843 (15.8)	477 (11.5)***	2,544 (17.2)	31 (8.9)***
Insulins	34,311 (27.3)	956 (23.1)***	3,870 (26.1)	39 (11.2)***

© 2024 Chuang M-H et al. *JAMA Network Open.*

Beta blockers	31,563 (25.2)	676 (16.3)***	3,082 (20.8)	32 (9.2)***
ACE inhibitors	25,812 (20.6)	613 (14.8)***	2,871 (19.4)	42 (12.0)***
Angiotensin II receptor blockers	27,153 (21.6)	552 (13.3)***	2,628 (17.7)	28 (8.0)***
Diuretics	35,796 (28.5)	771 (18.6)***	3,775 (25.5)	45 (12.9)***
Calcium channel blockers	24,569 (19.6)	528 (12.8)***	2,298 (15.5)	26 (7.4)***
**Laboratory tests**, mean (SD)				
Hemoglobin A1c, %	8.01 (1.95)	8.39 (2.18)***	7.66 (1.82)	8.26 (2.26)***
Hemoglobin, mg/dL	13.4 (1.9)	13.3 (2.1)	13.7 (1.8)	14.1 (1.6)*
eGFR, mL/min/1.73m^2	80.3 (27.7)	83.1 (30.3)***	81.4 (24.9)	87.8 (25.6)***
SBP, mmHg	131.8 (17.9)	132.0 (9.1)	131.4 (17.1)	130.0 (14.9)
LDL cholesterol, mg/dL	92.3 (38.6)	98.4 (38.9)***	92.9 (37.3)	103.1 (38.4)***
Total cholesterol, mg/dL	169.3 (49.2)	172.0 (50.3)	169.0 (50.8)	173.2 (48.4)
BMI, kg/m^2	34.8 (6.7)	35.0 (6.8)	36.7 (6.2)	36.7 (6.3)

*: p <0.05, **: p <0.01, ***: p <0.001 compared with the included group.

† Patient counts for rare circumstances or diseases are obfuscated up to 10 by the database platform to safeguard protected health information that could lead to identification of certain individuals.

Abbreviations: ACE, angiotensin converting enzyme; BMI, body mass index; DPP-4, dipeptidyl peptidase-4; eGFR, estimated glomerular filtration rate; HIV, human immunodeficiency virus; HMG CoA, hydroxymethylglutaryl-CoA; LDL, low-density lipoprotein; SD, standard deviation; SES, socioeconomic status; SGLT2, sodium-glucose cotransporter 2.

© 2024 Chuang M-H et al. *JAMA Network Open.*

eTable 5. Adjusted Hazard Ratios for the Components of Major Adverse Cardiovascular and Kidney Events

Outcomes	No. of patients with outcome		Adjusted hazard ratio (95% CI)	*p*-value
	Tirzepatide (n=14,832)	GLP-1 RA (n=14,832)		
**Major adverse cardiovascular events**				
Myocardial infarction	230	286	0.81 (0.68–0.97)	0.02
Ischemic/hemorrhagic stroke	284	363	0.78 (0.67–0.91)	0.002
Cardiac death	33	36	0.91 (0.56–1.45)	0.68
**Major adverse kidney events**				
Stage 5 chronic kidney disease	16	35	0.45 (0.25–0.82)	0.01
End-stage renal disease	55	111	0.49 (0.35–0.67)	<0.001

Abbreviations: CI, confidence interval; GLP-1 RAs, Glucagon-like peptide-1 receptor agonists.

eTable 6. Sensitivity Analysis Incorporating Individuals Without Follow-Up Into Propensity Score Matching and Survival Analysis of Primary and Secondary Outcomes

Outcomes	Main analysis		Sensitivity analysis	
	aHR (95%CI)	*p*-value	aHR (95%CI)	*p*-value
No. of patients in each group, post-PSM	14,832		15,181	
**Primary outcome**				
All-cause mortality	0.58 (0.45–0.75)	<0.001	0.52 (0.40–0.67)	<0.001
**Secondary outcomes**				
MACE	0.80 (0.71–0.91)	<0.001	0.83 (0.73–0.93)	<0.001
MACE and all-cause mortality	0.76 (0.68–0.84)	<0.001	0.75 (0.67–0.84)	<0.001
Kidney events	0.52 (0.37–0.73)	<0.001	0.53 (0.38–0.74)	<0.001
AKI	0.78 (0.70–0.88)	<0.001	0.82 (0.73–0.92)	<0.001
MAKE	0.54 (0.44–0.67)	<0.001	0.51 (0.41–0.64)	<0.001

Abbreviations: aHR, adjusted hazard ratio; CI, confidence interval; GLP-1 RAs, Glucagon-like peptide-1 receptor agonists; MACE, major adverse cardiovascular events; MAKE, major adverse kidney events; PSM, propensity score matching.

eTable 7. Changes in Mean Glycated Hemoglobin Level and Body Weight (95% CI)

Times points	Glycated hemoglobin level, %		Body weight, kg	
	Tirzepatide	GLP-1 RA	Tirzepatide	GLP-1 RA
4 months	–0.87 (–0.90 to –0.84)	–0.66 (–0.69 to –0.63)	–2.8 (–3.1 to –2.5)	–1.5 (–1.8 to –1.2)
8 months	–1.10 (–1.13 to –1.08)	–0.78 (–0.81 to –0.76)	–5.6 (–6.0 to –5.3)	–3.2 (–3.5 to –2.8)
12 months	–1.11 (–1.14 to –1.08)	–0.74 (–0.77 to –0.71)	–7.3 (–7.9 to –6.8)	–4.5 (–5.0 to –4.0)
16 months	–1.15 (–1.20 to –1.11)	–0.74 (–0.78 to –0.70)	–9.1 (–10.0 to –8.2)	–6.5 (–7.4 to –5.7)
20 months	–1.06 (–1.14 to –0.98)	–0.72 (–0.78 to –0.65)	–9.8 (–11.1 to –8.4)	–6.8 (–8.1 to –5.5)
TD*	–0.34 (–0.44 to –0.24), *p* <0.001		–2.9 (–4.8 to –1.1), *p*=0.002	

* Mean treatment difference at 20 months (95% CI).
Abbreviations: CI, confidence interval; GLP-1 RAs, Glucagon-like peptide-1 receptor agonists.

© 2024 Chuang M-H et al. *JAMA Network Open.*

eFigure 1. Changes in Glycated Hemoglobin Level and Body Weight From Baseline

1A. Changes in glycated hemoglobin level

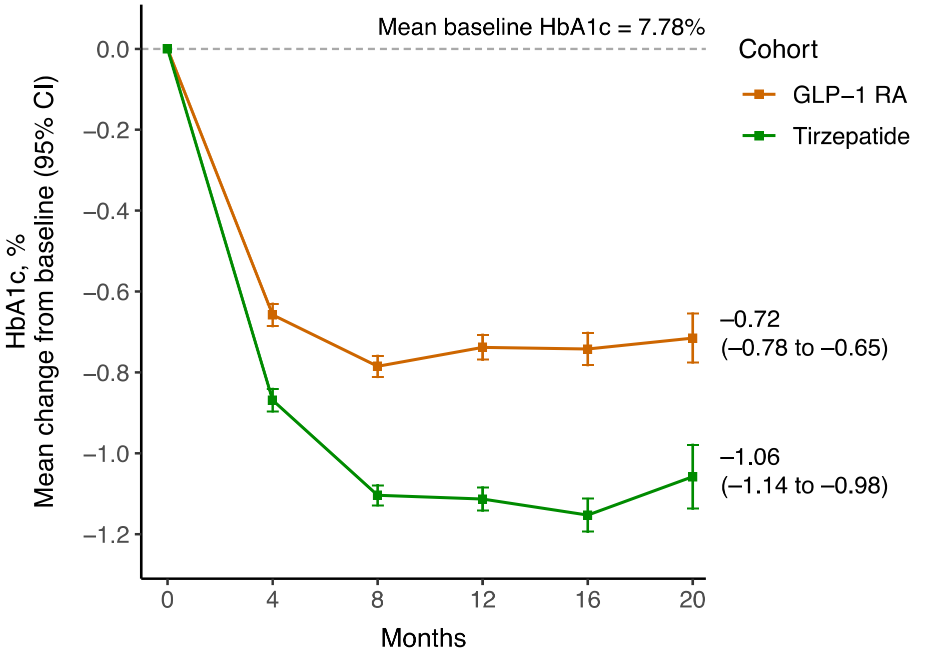

1B. Changes in body weight

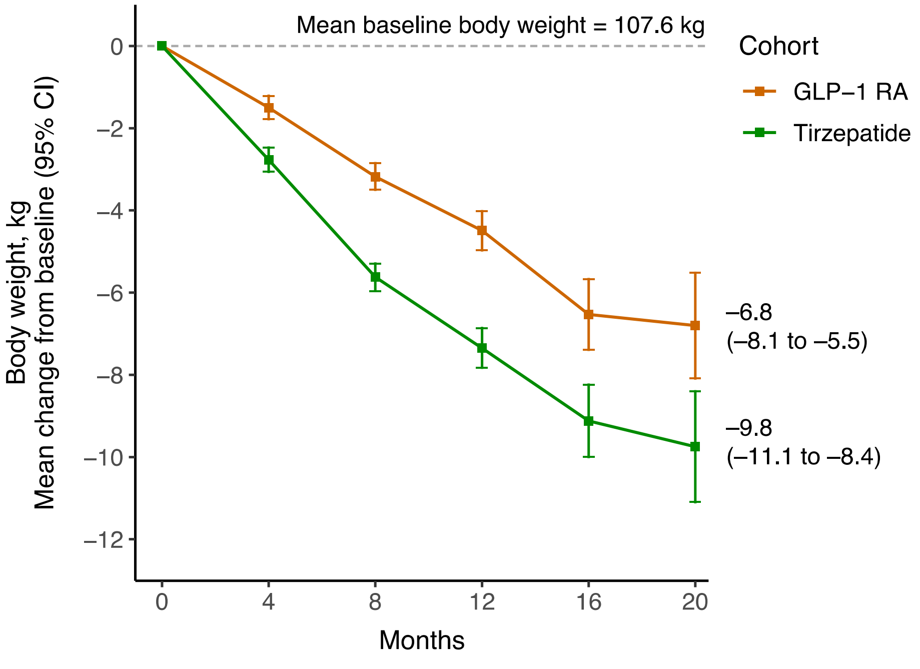

Abbreviations: CI, confidence interval; GLP-1 RA, glucagon-like peptide-1 receptor agonist.

© 2024 Chuang M-H et al. *JAMA Network Open.*

eFigure 2. Forest Plot of Primary and Secondary Outcomes Compared Between Different Doses of Tirzepatide and GLP-1 RA

2A. Tirzepatide compared to all GLP-1 RA or only those who received the maximal doses of GLP-1 RA

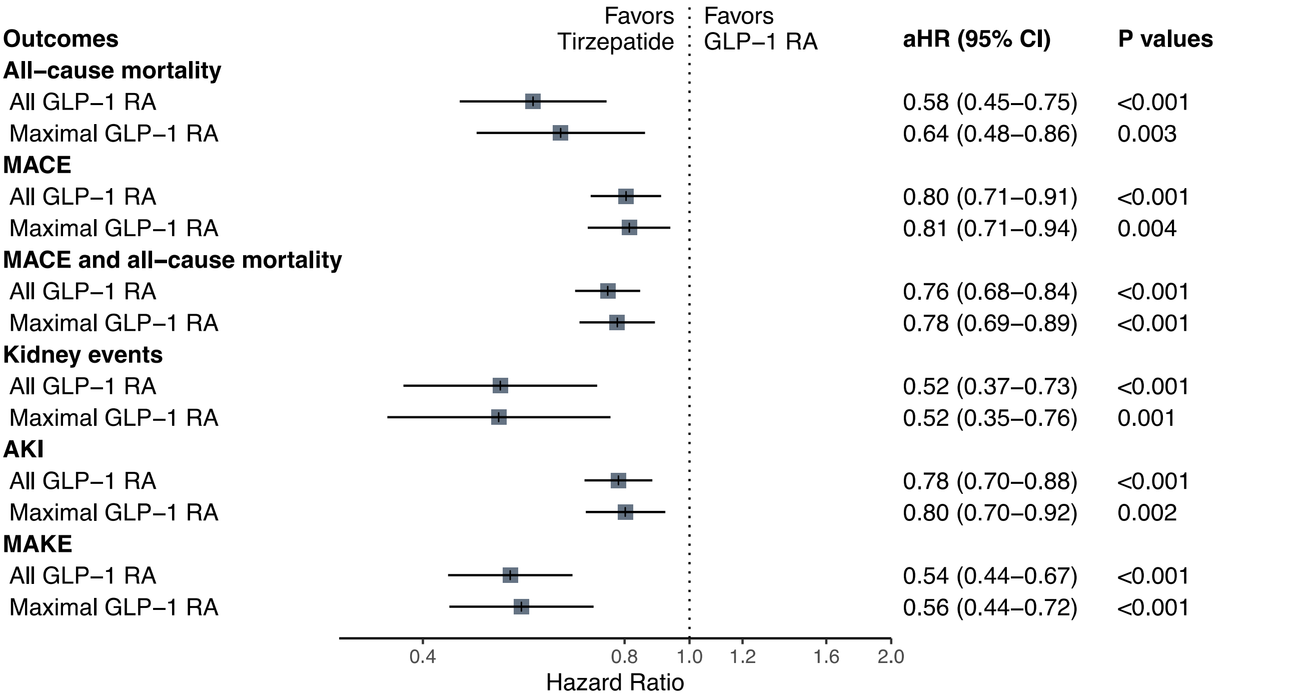

Abbreviations: aHR, adjusted hazard ratio; AKI, acute kidney injury; 95% CI, 95% confidence interval; GLP-1 RA, glucagon-like peptide-1 receptor agonist; MACE, major adverse cardiovascular events; MAKE, major adverse kidney events.

2B. Low-dose (maximum ≤7.5 mg) or high-dose (maximum ≥10 mg) tirzepatide compared to all GLP-RA

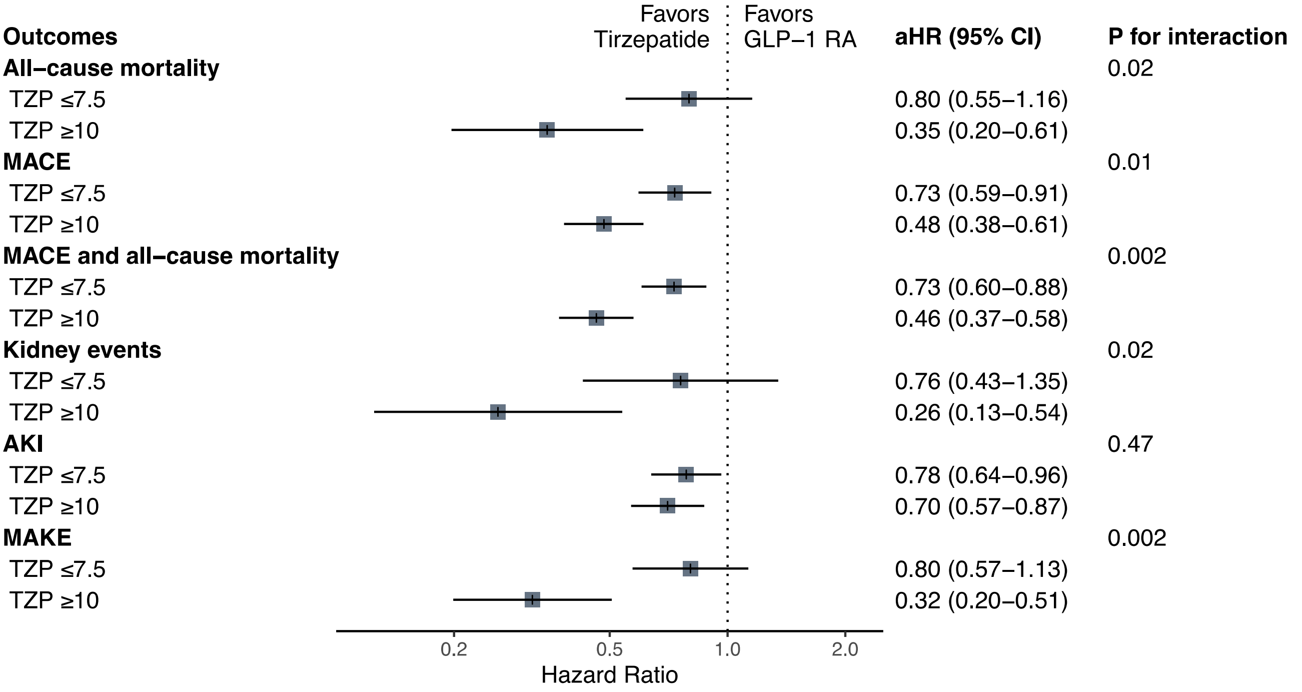

Abbreviations: aHR, adjusted hazard ratio; AKI, acute kidney injury; 95% CI, 95% confidence interval; GLP-1 RA, glucagon-like peptide-1 receptor agonist; MACE, major adverse cardiovascular events; MAKE, major adverse kidney events; TZP, tirzepatide.

© 2024 Chuang M-H et al. *JAMA Network Open.*

2C. Low-dose (maximum ≤7.5 mg) or high-dose (maximum ≥10 mg) tirzepatide compared to those who received the maximal doses of GLP1-RA

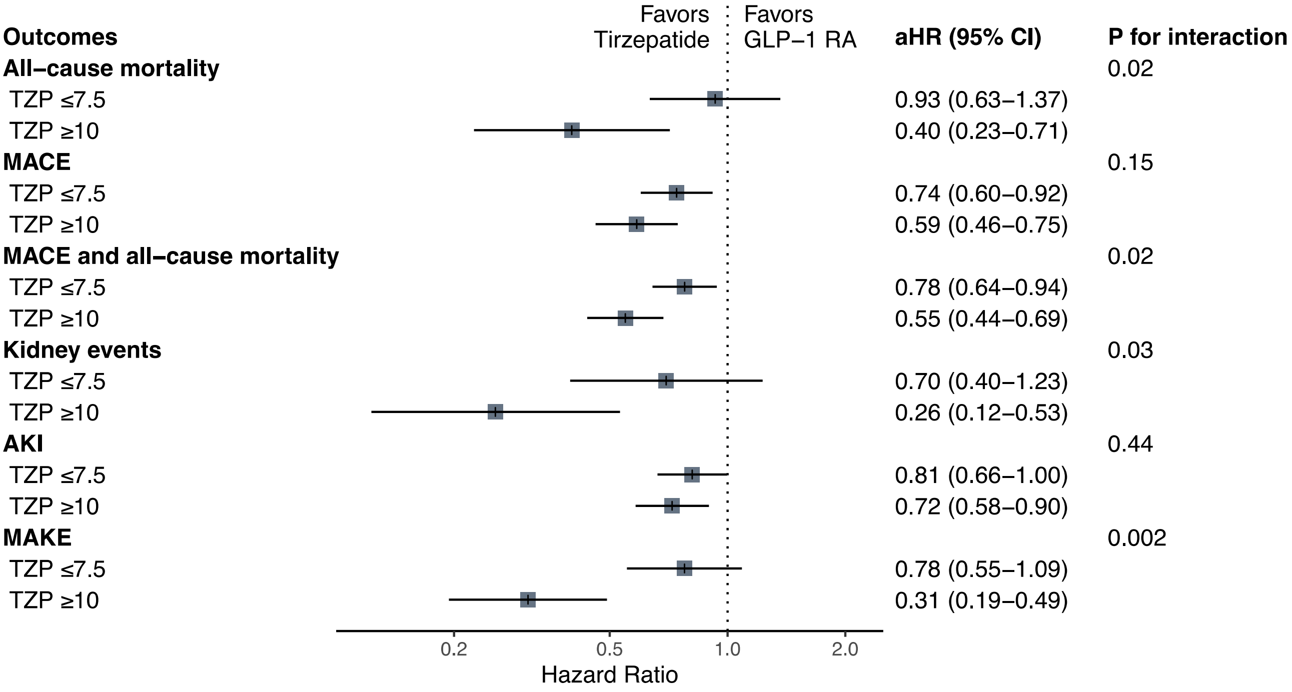

Abbreviations: aHR, adjusted hazard ratio; AKI, acute kidney injury; 95% CI, 95% confidence interval; GLP-1 RA, glucagon-like peptide-1 receptor agonist; MACE, major adverse cardiovascular events; MAKE, major adverse kidney events; TZP, tirzepatide.

eFigure 3. Results for Negative Control Outcomes and Analysis With Negative Control Exposure

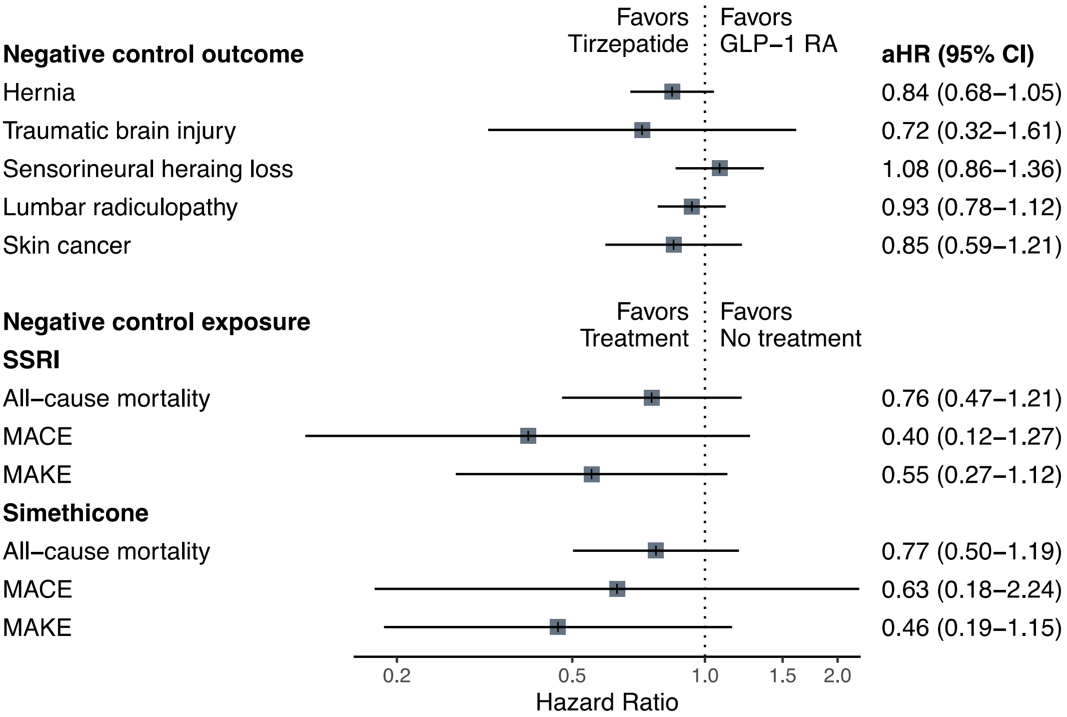

Abbreviations: aHR, adjusted hazard ratio; 95% CI, 95% confidence interval; GLP-1 RA, glucagon-like peptide-1 receptor agonist; MACE, major adverse cardiovascular events; MAKE, major adverse kidney events; SSRI, selective serotonin reuptake inhibitors.

© 2024 Chuang M-H et al. *JAMA Network Open.*

eFigure 4. Subgroup Analysis of All-Cause Mortality Between Tirzepatide and GLP-1 RA Groups

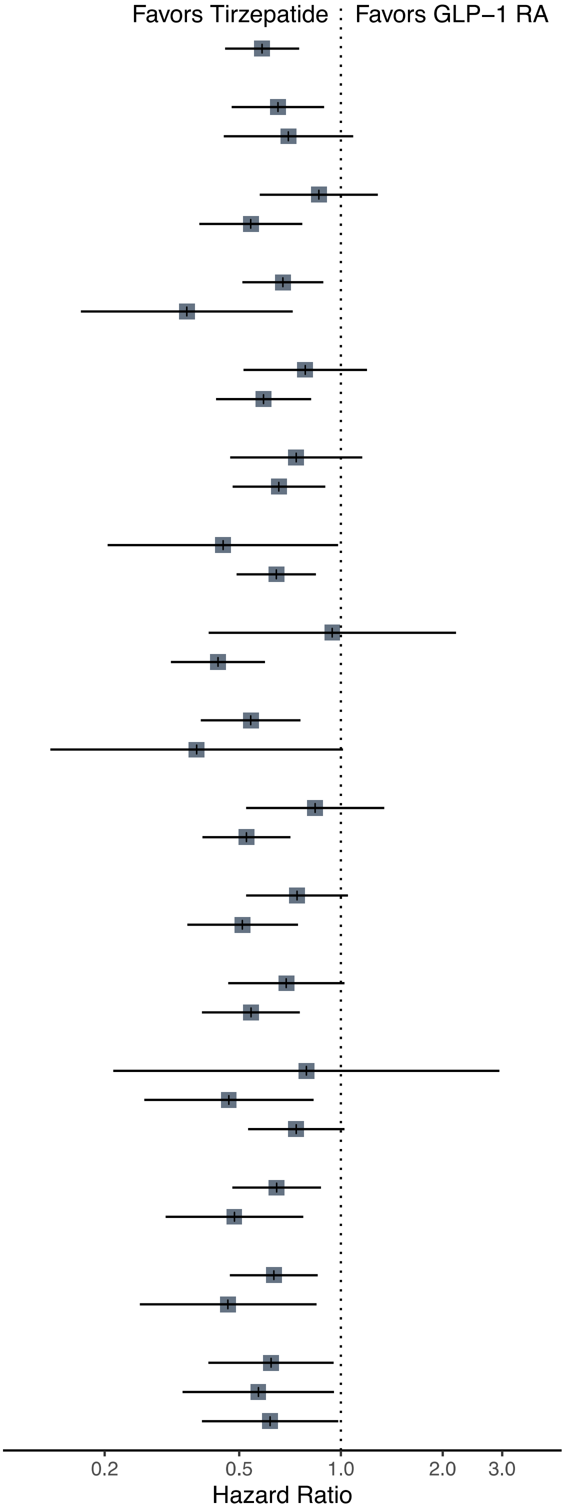

Subgroups	aHR (95% CI)	P for interaction
Overall	0.58 (0.45–0.75)	
**eGFR, mL/min/1.73m²**		0.80
≥45	0.65 (0.48–0.89)	
<45	0.70 (0.45–1.09)	
**HbA1c, %**		0.09
≥7	0.86 (0.58–1.29)	
<7	0.54 (0.38–0.77)	
**Hypertension**		0.10
HTN (+)	0.67 (0.51–0.89)	
HTN (–)	0.35 (0.17–0.72)	
**Ischemic heart diseases**		0.29
IHD (+)	0.78 (0.52–1.19)	
IHD (–)	0.59 (0.43–0.82)	
**Heart failure**		0.67
HF (+)	0.74 (0.47–1.16)	
HF (–)	0.66 (0.48–0.90)	
**Proteinuria**		0.39
Poteinuria (+)	0.45 (0.20–0.98)	
Proteinuria (–)	0.64 (0.49–0.84)	
**Metformin**		0.09
Metformin (+)	0.94 (0.41–2.19)	
Metformin (–)	0.43 (0.31–0.60)	
**Insulin**		0.49
Insulin (+)	0.54 (0.39–0.76)	
Insulin (–)	0.37 (0.14–1.01)	
**SGLT2i**		0.10
SGLT2i (+)	0.84 (0.52–1.34)	
SGLT2i (–)	0.53 (0.39–0.71)	
**ACEi/ARBs**		0.16
ACEi/ARB (+)	0.74 (0.52–1.05)	
ACEi/ARB (–)	0.51 (0.35–0.75)	
**Previous GLP–1 RA**		0.36
Ever user	0.69 (0.46–1.02)	
Never user	0.54 (0.39–0.76)	
**LDL, mg/dL**		0.39
≥160	0.79 (0.21–2.94)	
100–160	0.47 (0.26–0.83)	
<100	0.74 (0.53–1.02)	
**DM complications**		0.31
Complicaitons (+)	0.65 (0.48–0.87)	
Complications (–)	0.48 (0.30–0.77)	
**BMI, kg/m²**		0.36
≥30	0.63 (0.47–0.85)	
<30	0.46 (0.25–0.85)	
**Indication**		0.96
For DM	0.62 (0.41–0.95)	
For OW/OB	0.57 (0.34–0.95)	
For both	0.62 (0.39–0.98)	

Abbreviations: ACEi, angiotensin converting enzyme inhibitor; ARB, angiotensin II receptor blocker; aHR, adjusted hazard ratio; BMI, body mass index; 95% CI, 95% confidence interval; DM, diabetes mellitus; eGFR, estimated glomerular filtration rate; GLP-1 RA, glucagon-like peptide-1 receptor agonist; LDL, low-density lipoprotein cholesterol; MACE, major adverse cardiovascular events; MAKE, major adverse kidney events; OB, obesity; OW, overweight; SGLT2i, sodium-glucose cotransporter 2 inhibitors.

© 2024 Chuang M-H et al. *JAMA Network Open.*

eFigure 5. Subgroup Analysis of Major Adverse Cardiovascular Events Between Tirzepatide and GLP-1 RA Groups

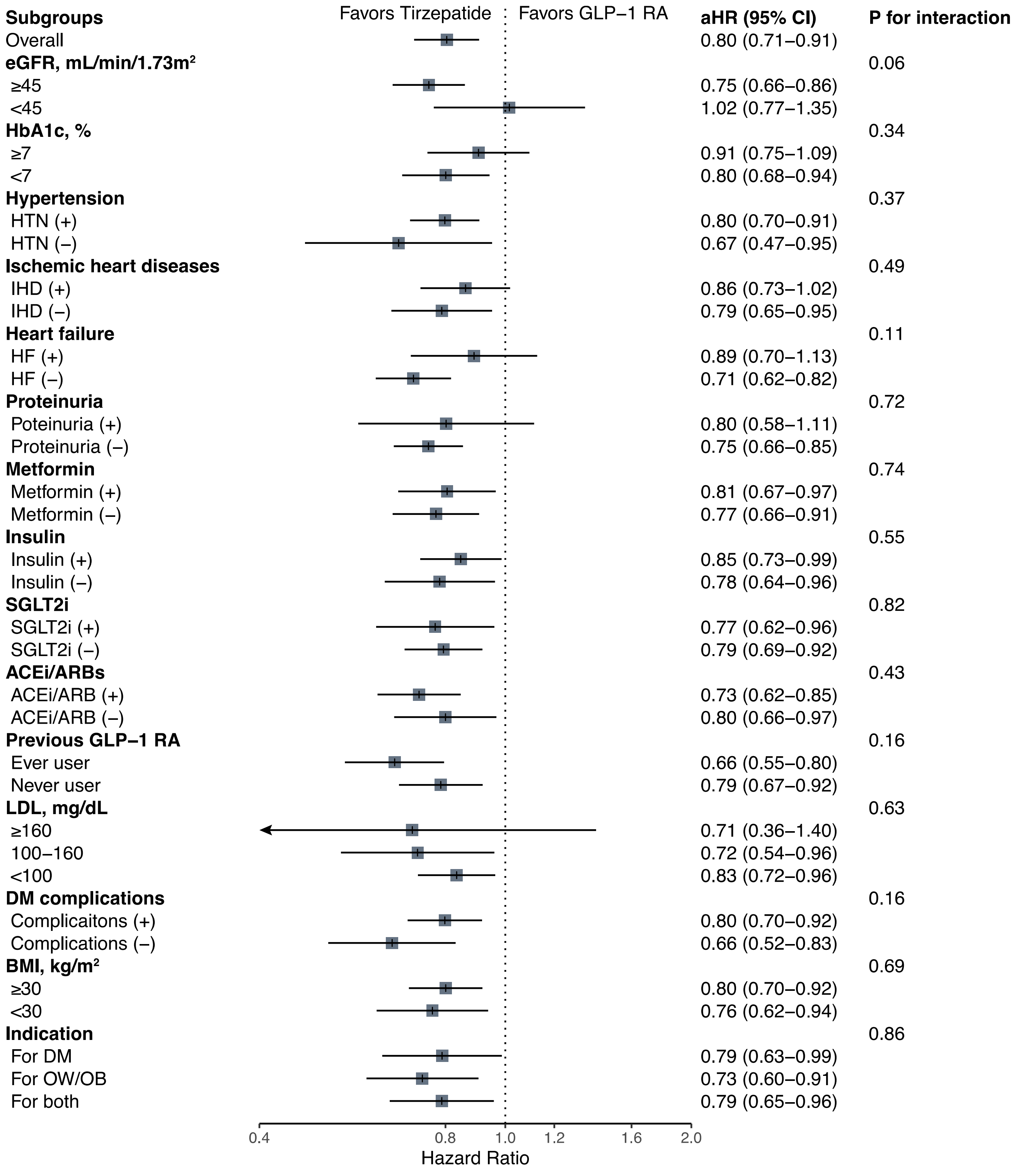

Abbreviations: ACEi, angiotensin converting enzyme inhibitor; ARB, angiotensin II receptor blocker; aHR, adjusted hazard ratio; BMI, body mass index; 95% CI, 95% confidence interval; DM, diabetes mellitus; eGFR, estimated glomerular filtration rate; GLP-1 RA, glucagon-like peptide-1 receptor agonist; LDL, low-density lipoprotein cholesterol; MACE, major adverse cardiovascular events; MAKE, major adverse kidney events; OB, obesity; OW, overweight; SGLT2i, sodium-glucose cotransporter 2 inhibitors.

© 2024 Chuang M-H et al. *JAMA Network Open.*

eFigure 6. Subgroup Analysis of the Composite of Major Adverse Cardiovascular Events and All-Cause Mortality Between Tirzepatide and GLP-1 RA Groups

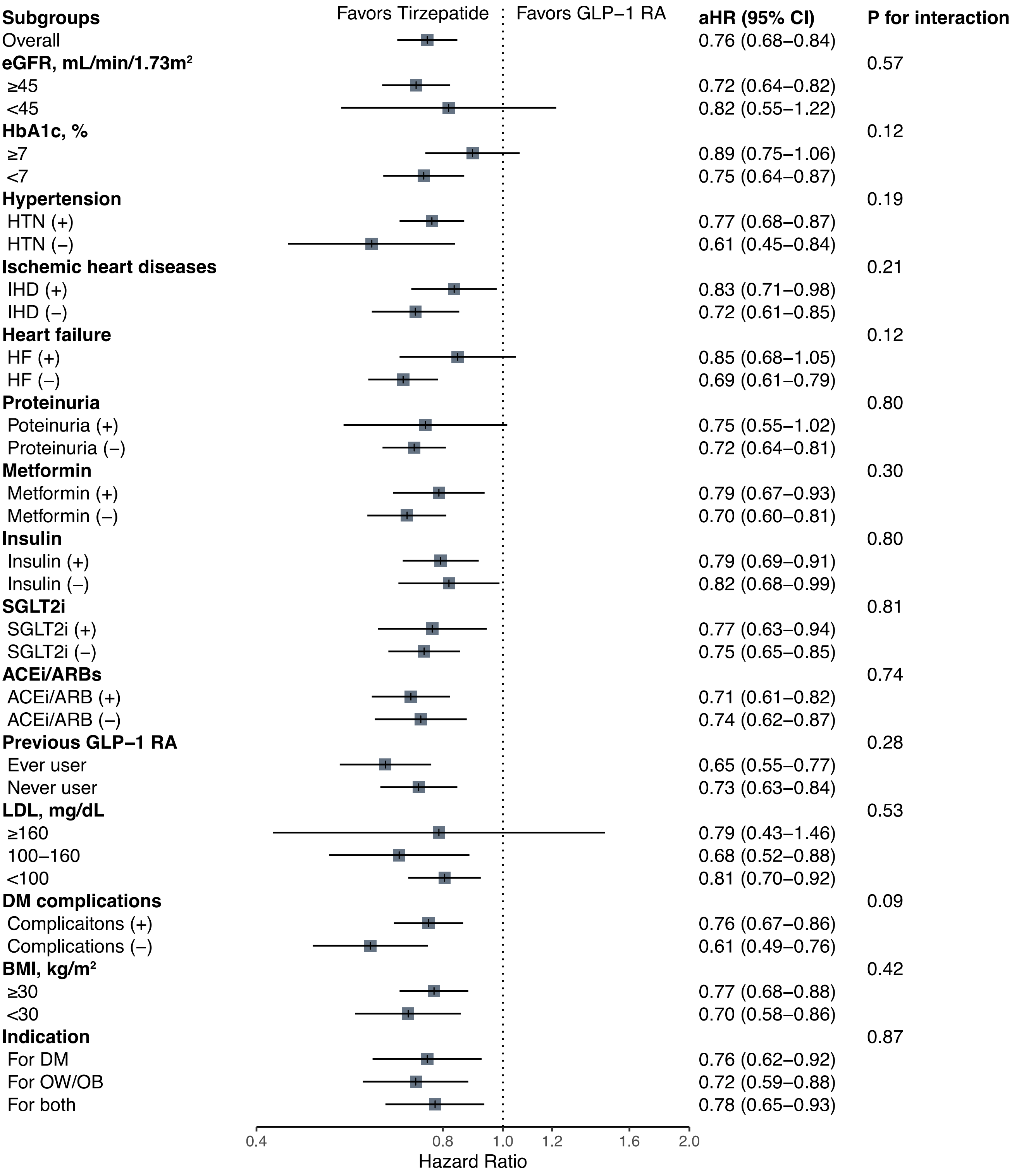

Abbreviations: ACEi, angiotensin converting enzyme inhibitor; ARB, angiotensin II receptor blocker; aHR, adjusted hazard ratio; BMI, body mass index; 95% CI, 95% confidence interval; DM, diabetes mellitus; eGFR, estimated glomerular filtration rate; GLP-1 RA, glucagon-like peptide-1 receptor agonist; LDL, low-density lipoprotein cholesterol; MACE, major adverse cardiovascular events; MAKE, major adverse kidney events; OB, obesity; OW, overweight; SGLT2i, sodium-glucose cotransporter 2 inhibitors.

© 2024 Chuang M-H et al. *JAMA Network Open.*

eFigure 7. Subgroup Analysis of Kidney Events Between Tirzepatide and GLP-1 RA Groups

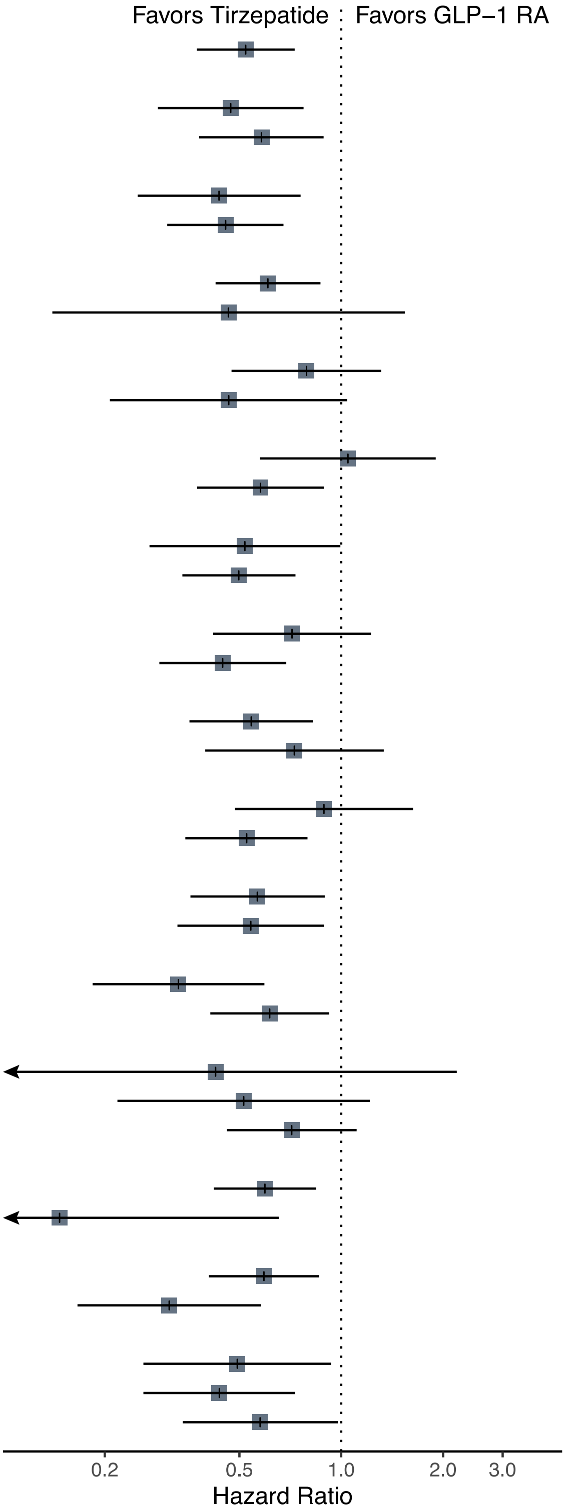

**Subgroups**	**aHR (95% CI)**	**P for interaction**
Overall	0.52 (0.37–0.73)	
**eGFR, mL/min/1.73m²**		0.53
≥45	0.47 (0.29–0.77)	
<45	0.58 (0.38–0.89)	
**HbA1c, %**		0.90
≥7	0.44 (0.25–0.76)	
<7	0.46 (0.31–0.68)	
**Hypertension**		0.67
HTN (+)	0.61 (0.43–0.87)	
HTN (−)	0.46 (0.14–1.54)	
**Ischemic heart diseases**		0.28
IHD (+)	0.79 (0.47–1.31)	
IHD (−)	0.47 (0.21–1.04)	
**Heart failure**		0.11
HF (+)	1.05 (0.58–1.90)	
HF (−)	0.58 (0.38–0.89)	
**Proteinuria**		0.92
Poteinuria (+)	0.52 (0.27–0.99)	
Proteinuria (−)	0.50 (0.34–0.73)	
**Metformin**		0.18
Metformin (+)	0.72 (0.42–1.22)	
Metformin (−)	0.45 (0.29–0.69)	
**Insulin**		0.44
Insulin (+)	0.54 (0.36–0.82)	
Insulin (−)	0.73 (0.40–1.34)	
**SGLT2i**		0.16
SGLT2i (+)	0.89 (0.49–1.63)	
SGLT2i (−)	0.53 (0.35–0.80)	
**ACEi/ARBs**		0.90
ACEi/ARB (+)	0.57 (0.36–0.89)	
ACEi/ARB (−)	0.54 (0.33–0.89)	
**Previous GLP−1 RA**		0.09
Ever user	0.33 (0.18–0.59)	
Never user	0.61 (0.41–0.92)	
**LDL, mg/dL**		0.70
≥160	0.43 (0.08–2.19)	
100–160	0.52 (0.22–1.22)	
<100	0.71 (0.46–1.11)	
**DM complications**		0.07
Complicaitons (+)	0.60 (0.42–0.84)	
Complications (−)	0.15 (0.03–0.65)	
**BMI, kg/m²**		0.08
≥30	0.59 (0.41–0.86)	
<30	0.31 (0.17–0.58)	
**Indication**		0.76
For DM	0.49 (0.26–0.93)	
For OW/OB	0.44 (0.26–0.73)	
For both	0.58 (0.34–0.98)	

Abbreviations: ACEi, angiotensin converting enzyme inhibitor; ARB, angiotensin II receptor blocker; aHR, adjusted hazard ratio; BMI, body mass index; 95% CI, 95% confidence interval; DM, diabetes mellitus; eGFR, estimated glomerular filtration rate; GLP-1 RA, glucagon-like peptide-1 receptor agonist; LDL, low-density lipoprotein cholesterol; MACE, major adverse cardiovascular events; MAKE, major adverse kidney events; OB, obesity; OW, overweight; SGLT2i, sodium-glucose cotransporter 2 inhibitors.

© 2024 Chuang M-H et al. *JAMA Network Open.*

eFigure 8. Subgroup Analysis of Acute Kidney Injury Between Tirzepatide and GLP-1 RA Groups

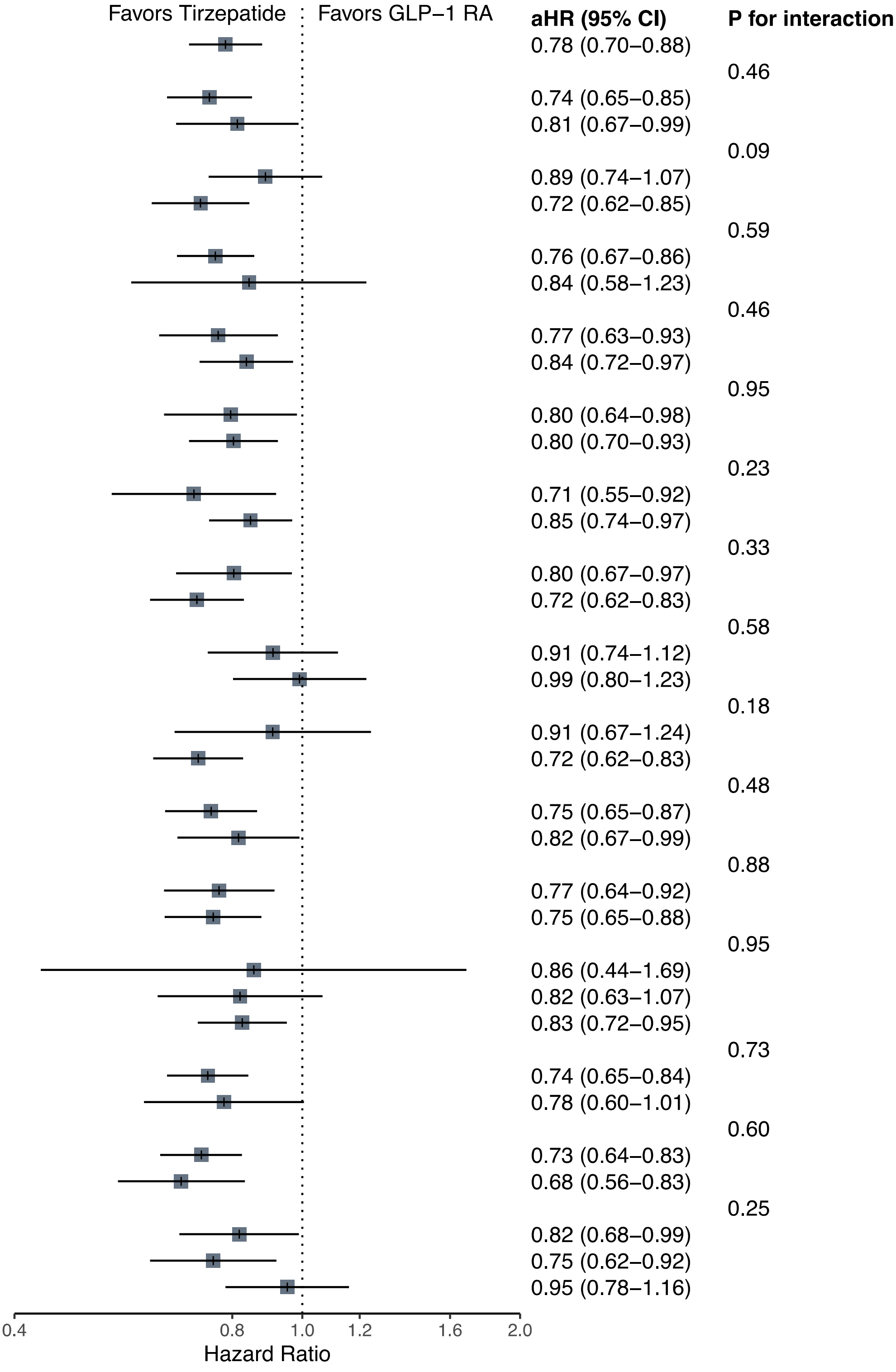

Subgroups	aHR (95% CI)	P for interaction
Overall	0.78 (0.70–0.88)	
**eGFR, mL/min/1.73m²**		0.46
≥45	0.74 (0.65–0.85)	
<45	0.81 (0.67–0.99)	
**HbA1c, %**		0.09
≥7	0.89 (0.74–1.07)	
<7	0.72 (0.62–0.85)	
**Hypertension**		0.59
HTN (+)	0.76 (0.67–0.86)	
HTN (−)	0.84 (0.58–1.23)	
**Ischemic heart diseases**		0.46
IHD (+)	0.77 (0.63–0.93)	
IHD (−)	0.84 (0.72–0.97)	
**Heart failure**		0.95
HF (+)	0.80 (0.64–0.98)	
HF (−)	0.80 (0.70–0.93)	
**Proteinuria**		0.23
Poteinuria (+)	0.71 (0.55–0.92)	
Proteinuria (−)	0.85 (0.74–0.97)	
**Metformin**		0.33
Metformin (+)	0.80 (0.67–0.97)	
Metformin (−)	0.72 (0.62–0.83)	
**Insulin**		0.58
Insulin (+)	0.91 (0.74–1.12)	
Insulin (−)	0.99 (0.80–1.23)	
**SGLT2i**		0.18
SGLT2i (+)	0.91 (0.67–1.24)	
SGLT2i (−)	0.72 (0.62–0.83)	
**ACEi/ARBs**		0.48
ACEi/ARB (+)	0.75 (0.65–0.87)	
ACEi/ARB (−)	0.82 (0.67–0.99)	
**Previous GLP−1 RA**		0.88
Ever user	0.77 (0.64–0.92)	
Never user	0.75 (0.65–0.88)	
**LDL, mg/dL**		0.95
≥160	0.86 (0.44–1.69)	
100–160	0.82 (0.63–1.07)	
<100	0.83 (0.72–0.95)	
**DM complications**		0.73
Complicaitons (+)	0.74 (0.65–0.84)	
Complications (−)	0.78 (0.60–1.01)	
**BMI, kg/m²**		0.60
≥30	0.73 (0.64–0.83)	
<30	0.68 (0.56–0.83)	
**Indication**		0.25
For DM	0.82 (0.68–0.99)	
For OW/OB	0.75 (0.62–0.92)	
For both	0.95 (0.78–1.16)	

Abbreviations: ACEi, angiotensin converting enzyme inhibitor; ARB, angiotensin II receptor blocker; aHR, adjusted hazard ratio; BMI, body mass index; 95% CI, 95% confidence interval; DM, diabetes mellitus; eGFR, estimated glomerular filtration rate; GLP-1 RA, glucagon-like peptide-1 receptor agonist; LDL, low-density lipoprotein cholesterol; MACE, major adverse cardiovascular events; MAKE, major adverse kidney events; OB, obesity; OW, overweight; SGLT2i, sodium-glucose cotransporter 2 inhibitors.

© 2024 Chuang M-H et al. *JAMA Network Open.*

eFigure 9. Subgroup Analysis of Major Adverse Kidney Events Between Tirzepatide and GLP-1 RA Groups

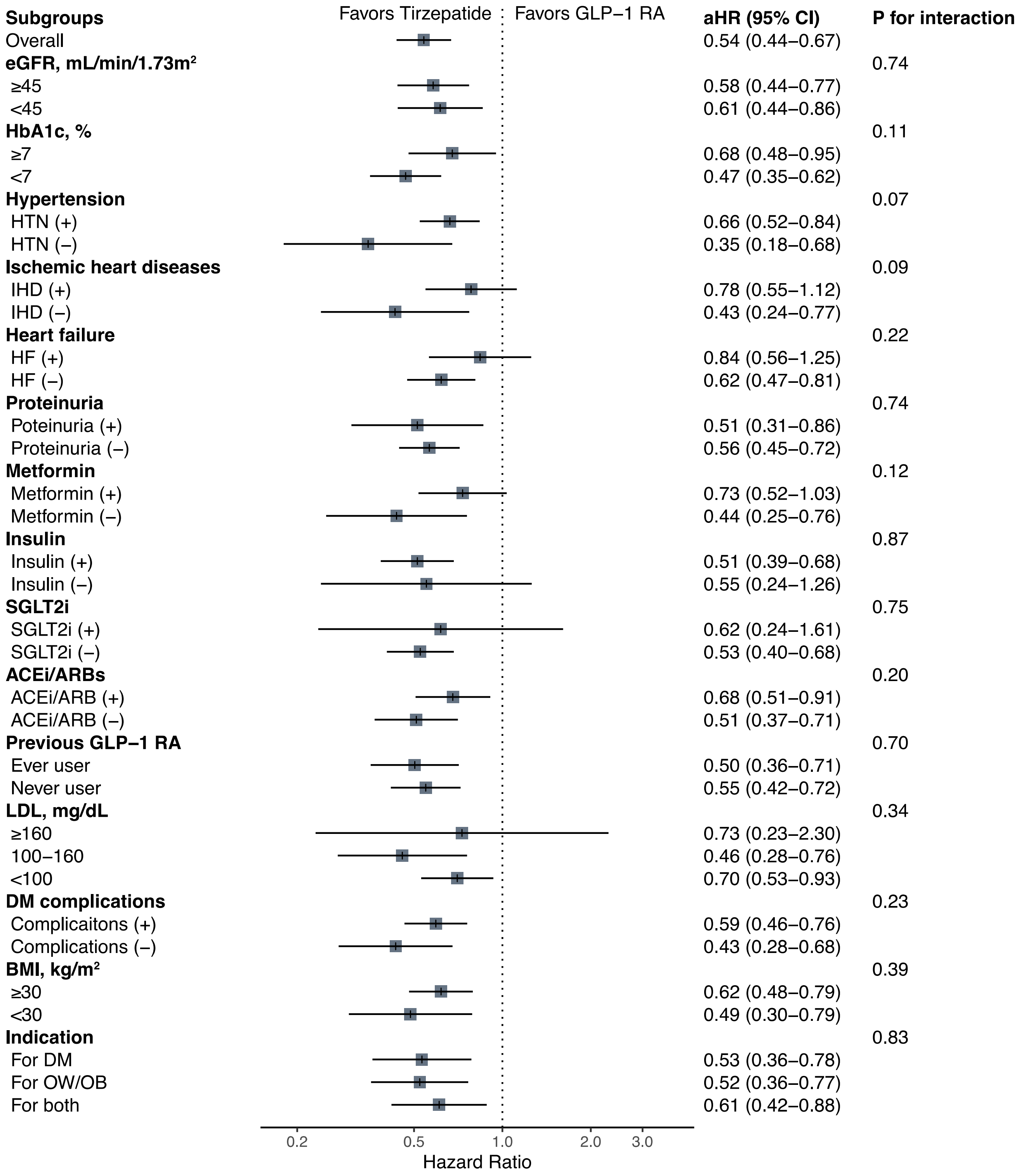

Abbreviations: ACEi, angiotensin converting enzyme inhibitor; ARB, angiotensin II receptor blocker; aHR, adjusted hazard ratio; BMI, body mass index; 95% CI, 95% confidence interval; DM, diabetes mellitus; eGFR, estimated glomerular filtration rate; GLP-1 RA, glucagon-like peptide-1 receptor agonist; LDL, low-density lipoprotein cholesterol; MACE, major adverse cardiovascular events; MAKE, major adverse kidney events; OB, obesity; OW, overweight; SGLT2i, sodium-glucose cotransporter 2 inhibitors.

© 2024 Chuang M-H et al. *JAMA Network Open.*
