# Supplementary material for: Clinical Outcomes of Tirzepatide or GLP-1 Receptor Agonists in Individuals With Type 2 Diabetes
Source: JAMA Netw Open. 2024 Aug 12;7(8):e2427258. doi: 10.1001/jamanetworkopen.2024.27258 (PMC11320168; doi:10.1001/jamanetworkopen.2024.27258)
Supplement: Supplement 2. — Data Sharing Statement [file jamanetwopen-e2427258-s002.pdf]

Data Sharing Statement

Chuang. Clinical Outcomes of Tirzepatide or GLP-1 Receptor Agonists in Individuals With Type 2 Diabetes. *JAMA Netw Open*. Published August 12, 2024.
doi:10.1001/jamanetworkopen.2024.27258

Data
Data available: No
